# Integrating EEG and EMG data: a novel statistical pipeline for investigating brain-muscle interaction in experimental neuroarchaeology

**DOI:** 10.1007/s00429-025-02961-1

**Published:** 2025-06-16

**Authors:** Simona Affinito, Brienna Eteson, Fotios Alexandros Karakostis

**Affiliations:** 1 Department of Geosciences, DFG Center for Advanced Studies “Words, Bones, Genes, Tools”, Eberhard Karls University of Tübingen, Tübingen, Germany; 2 Department of Geosciences, Paleoanthropology, Senckenberg Centre for Human Evolution and Palaeoenvironment, Eberhard Karls University of Tübingen, Tübingen, Germany; 3 Integrative Prehistory and Archaeological Science, University of Basel, Basel, Switzerland

**Keywords:** Experimental neuroarchaeology, Electroencephalography (EEG), Electromyography (EMG), Tool use, Statistical pipeline, Hominin behavior

## Abstract

This study introduces a novel multivariate statistical pipeline for integrating and analyzing EEG and EMG data in experimental neuroarchaeology, providing a robust framework for investigating brain-hand interactions during a variety of tool-related behaviours. By implementing a multistep approach, it effectively characterizes task/condition-related variations while detecting meaningful covariation patterns between neural and muscular activity. Compared to traditional univariate techniques, this pipeline better addresses the complexity of multimodal datasets, offering a more comprehensive understanding of cognitive-motor dynamics. We demonstrate the utility of this approach using data from a previously conducted experiment on early hominin stone tool use, illustrating how electroencephalography (EEG) and electromyography (EMG) integration can reveal interactions between brain and hand processes across tasks of varying manual complexity. This methodological advancement not only enhances the study of tool-related behaviors but also establishes a standardized framework for future research in neuroarchaeology. Beyond stone tools, this approach could be extended to other forms of material culture, such as ornaments and engravings, contributing to a broader understanding of manual skill development and symbolic behavior.

**Supplementary Information:**

The online version contains supplementary material available at 10.1007/s00429-025-02961-1.

## Introduction

Understanding the cognitive abilities underlying complex human behaviors—such as tool use, tool making, and language—remains one of the greatest challenges in studying the evolution of hominin cognition. When the subjects of investigation are extinct species, researchers face significant limitations. Unlike modern species, extinct hominins’ behaviors cannot be observed in action, nor can their brain function be studied in vivo. Additionally, there is no direct anatomical correspondence to the brain itself, as brains are not preserved in the fossil record. As a result, any investigation in this area must rely on indirect evidence (e.g., archaeological artifacts, cranial endocast, modern human/non-human primate behaviors). The integration of different disciplines and techniques is essential to mitigate these limitations as much as possible, as no single approach can fully address these challenges. Together, interdisciplinary fields such as paleoneurology (Bruner 2017, 2019), cognitive archaeology (Wynn and Coolidge 2016; Bruner 2024), and neuroarchaeology (Stout and Hecht 2015; see also Bryche et al. 2024 for a recent review) can provide a more integrated view and help to develop a comprehensive understanding of the cognitive processes that supported early human behaviors.

One particularly promising approach is experimental neuroarchaeology, which combines methods from neuroscience and archaeology by using neuroimaging techniques in controlled experimental settings to identify the neural and cognitive correlates of behaviors inferred from the archaeological record (Stout 2023). For example, brain activity can be recorded during the performance of specific tasks that resemble those presumed to be performed by early hominins (e.g., using stone tools or making stone tools). By identifying brain regions activated by task-specific demands, we can make inferences about the cognitive skills required and the neural substrates likely involved in the emergence of novel behaviors in the course of hominin evolution (Salagnon et al. 2020). To date, various brain imaging techniques have been used in neuroarchaeology to investigate these relationships: functional magnetic resonance imaging (fMRI) (Stout et al. 2011, 2015), structural magnetic resonance imaging (sMRI) and diffusion magnetic resonance imaging (dMRI) (Hecht et al. 2015, 2023), positron emission tomography (PET) (Stout et al. 2000, 2008; Stout and Chaminade 2007), functional transcranial doppler sonography (fTCD) (Uomini and Meyer 2013), functional near-infrared spectroscopy (fNIRS) (Putt et al. 2017, 2019), and electroencephalography (EEG) (Affinito et al. 2024). The choice of technique depends on the specific research objectives and experimental constraints, as each method has its own advantages and limitations. For studies requiring high spatial resolution, fMRI and PET have been commonly used. fMRI enables precise, non-invasive localization of brain activity but has low temporal resolution and is highly sensitive to motion artifacts, limiting its use in studies involving movement (Logothetis 2008; Salagnon et al. 2020). PET, on the other hand, has significant drawbacks. It requires the injection of a radiotracer, raising safety concerns and complicating participant recruitment in non-clinical research settings such as neuroarchaeology. For motor-related experiments, EEG and fNIRS provide more practical alternatives, especially given their availability in portable, cost-effective systems. EEG, with its millisecond-level temporal resolution, is ideal for real-time neural activity monitoring and is well-suited for integration with other biosignals, making it useful for multimodal and real-time dynamic studies and allowing for the analysis of brain activity during separate stages of each tool-related action (e.g., holding, aiming, and execution during tool-use). Its spatial resolution can be improved with high-density EEG systems (Marino and Mantini 2024), but it remains susceptible to movement artifacts. fNIRS represents a good compromise between EEG and fMRI, as it provides better spatial resolution than EEG and better temporal resolution than fMRI, while also being less susceptible to movement artifacts. However, its temporal resolution remains lower than EEG, and its spatial resolution and penetration depth are more restricted than fMRI.

Tool-related behaviors, such as stone tool manufacture and use, have been a major focus in experimental neuroarchaeology due to the crucial role technological innovation has played in human evolution. The trend of encephalization observed in the genus *Homo* appears to align with the increasing complexity of stone tools, suggesting a potential link between cognitive development and technological advancements (Stout et al. 2008). This connection highlights the importance of studying brain-tool interaction. Similarly, experimental archaeology has explored hand-tool interaction, focusing on the biomechanical and functional demands of tool-related behaviors to understand how these activities may have influenced hand function and manipulative abilities (e.g., Marzke and Shackley 1986; see also review from Kivell et al. 2023). Techniques such as electromyography (EMG), which tracks real-time muscle activation, and kinematic analysis, which studies movement dynamics, have provided valuable insights into these aspects of tool knapping and use (e.g., Hamrick et al. 1998; Marzke et al. 1998; Williams et al. 2010; Rolian et al. 2011; Key et al. 2020, 2021; Macchi et al. 2021). Despite important contributions from both fields, research on brain-hand-tool interaction remains largely fragmented. Studies have typically examined either the neural correlates of stone tool use/production or the biomechanical demands placed on the hand, but never have these dimensions been experimentally integrated within archaeological research.

To address this gap, we have previously published an open-access protocol that combines EEG and surface electromyography (sEMG) to study brain–hand interaction in real-time, allowing for the simultaneous recording of neural activity and muscle recruitment during tool-related behaviors (Eteson et al. 2024a, 2025). Even though this protocol enables the synchronous analysis of these signals, our previous studies have examined neural and biomechanical aspects separately (Affinito et al. 2024; Eteson et al. 2024b) rather than integrating them within the same multivariate analysis. Building on this framework, the current study aims to propose an original statistical pipeline for integrating and analyzing EEG and EMG data together.

EEG measures the electrical activity (electrical fields) generated by the synchronous activation of groups of neurons. Therefore, it provides direct and temporally precise information about neural activity and brain dynamics. This signal is expressed as rhythmic oscillations that can be decomposed into distinct frequency bands, each associated with different functional states. However, changes in the oscillatory pattern do not reflect more or less brain activity in a simplistic sense; rather, they represent shifts in the cognitive and functional state, shaping how information is processed. For example, modulations in beta-band activity are associated with motor planning (Turella et al. 2016), visual attention (Gola et al. 2013), and sensorimotor integration (Kilavik et al. 2013). Therefore, EEG activity is interpreted within a task-dependent framework, considering both the cortical origin of the signal and the functional context. By situating EEG findings within this framework, it is possible to understand how specific patterns of brain activity relate to cognitive functions and processes such as decision-making, ultimately allowing to make inferences about cognition and behavior. Although the relationship between neural activity and muscle recruitment pattern is complex and not yet fully understood, this interaction is likely not fixed. Recent studies have shown that motor cortical activity patterns vary depending on the task, even when involving the same limbs/muscles (Warriner et al. 2022), indicating that brain-muscle coordination is likely task-dependent. In this context, our integrative approach can offer a framework for directly examining and comparing brain-muscle interactions across tasks. In addition, differences in motor cortical covariation have also been observed between movement preparation and execution (Kaufman et al. 2014; Elsayed et al. 2016), which further highlights the utility of our approach, as the high temporal resolution allows us to track these interactions not only across tasks, but also between distinct stages of action.

In the past decade, the integration of EEG and EMG data has received increasing attention across different fields, especially in motor rehabilitation, prosthetics, and sport science (e.g., see review by Brambilla et al. 2021; Schneider et al. 2013; Yu et al. 2025). This integration has been implemented in various ways: in some cases, EEG and EMG are analyzed jointly within a single computational framework, such as coherence analysis or machine learning models (e.g., Guerrero-Mendez and Ruiz-Olaya 2022; Cho et al. 2022); in others, the two signals are processed separately and compared retrospectively, for example by aligning event-related responses and correlating time-varying features across modalities (e.g., Li et al. 2018). While many studies have primarily focused on assessing corticomuscular interaction, often through EEG–EMG coherence, fewer have expanded their scope to investigate broader aspects of sensorimotor or cognitive-motor integration.

Our aim is to propose an approach that may be better suited to supporting the integration of biomechanical insights from experimental archaeology with the cognitive and neural dimensions addressed in experimental neuroarchaeology. Specifically, we are interested in offering a framework that can account for the embodied nature of brain-muscle dynamics, without relying on strong model assumptions or task designs oriented primarily toward predictive performance.

To this end, our approach can help identify task-relevant, distributed patterns of covariation across brain and muscle activity. From an applied perspective, it may support investigations into whether biomechanical demands are reflected at the cognitive level. For instance, it could facilitate neuromechanical comparisons across tasks involving different types of stone tools that require distinct grasping patterns (e.g., handaxes, thumb scrapers, or hafted tools), as well as between key lithic reduction techniques that have been proposed to differ in motor and cognitive complexity (e.g., bipolar vs. free-hand knapping, or Mousterian vs. blade-like production strategies). Similarly, it could be used to compare neural and muscular dynamics (and their interaction) before and after training in tool production (or use), in order to examine how skill acquisition influences the relationship between muscular efficiency and cognitive functions.

Archaeological studies using EMG typically rely on univariate statistical approaches to analyze muscle recruitment and activation. Commonly used univariate techniques include t tests and analysis of variance (ANOVA), as well as non-parametric tests such as the Kruskal–Wallis or Mann–Whitney U, which help identify and assess statistically significant differences between groups or tasks (Hamrick et al. 1998; Marzke et al. 1998; Key et al. 2020, 2021; Macchi et al. 2021). For more complex datasets involving multiple variables, such as the combination of kinematic and EMG data, multivariate methods like principal component analysis (PCA) and statistical parametric mapping (SPM) have been used (Macchi et al. 2021). In neuroarchaeology, MRI analyses often use general linear models (GLM) to detect significant clusters (Stout et al. 2011, 2015; Hecht et al. 2015, 2023). As with EMG analyses, to compare and evaluate differences between conditions and groups, researchers employ statistical tests such as t tests and ANOVA, along with non-parametric tests (e.g., Wilcoxon signed-rank, Mann–Whitney U) (e.g., Stout and Chaminade 2007; Stout et al. 2015; Putt et al. 2017, 2019; Affinito et al. 2024) and permutation-based methods (e.g., Hecht et al. 2015, 2023).

Here we introduce a multivariate statistical pipeline that integrates EEG and EMG data to investigate brain-hand interactions during human-like stone tool use and production tasks. Our approach focuses on three main objectives: (1) selecting the most relevant EEG and EMG channels, (2) identifying patterns of variation across different experimental conditions, and (3) examining the covariations between neural and muscular activity. We demonstrate the pipeline using an example dataset from an early hominin stone tool use experiment we conducted, where previous work had analyzed EEG and EMG data separately, with insightful results (Affinito et al. 2024; Eteson et al. 2024b). By unifying these data, our approach can offer a currently missing tool for exploring how cognitive (brain) and biomechanical (hand) processes intersect across tasks of varying levels of technological and manual complexity.

## Materials and methods

### Statistical pipeline for multimodal data analysis

**Fig. 1:**
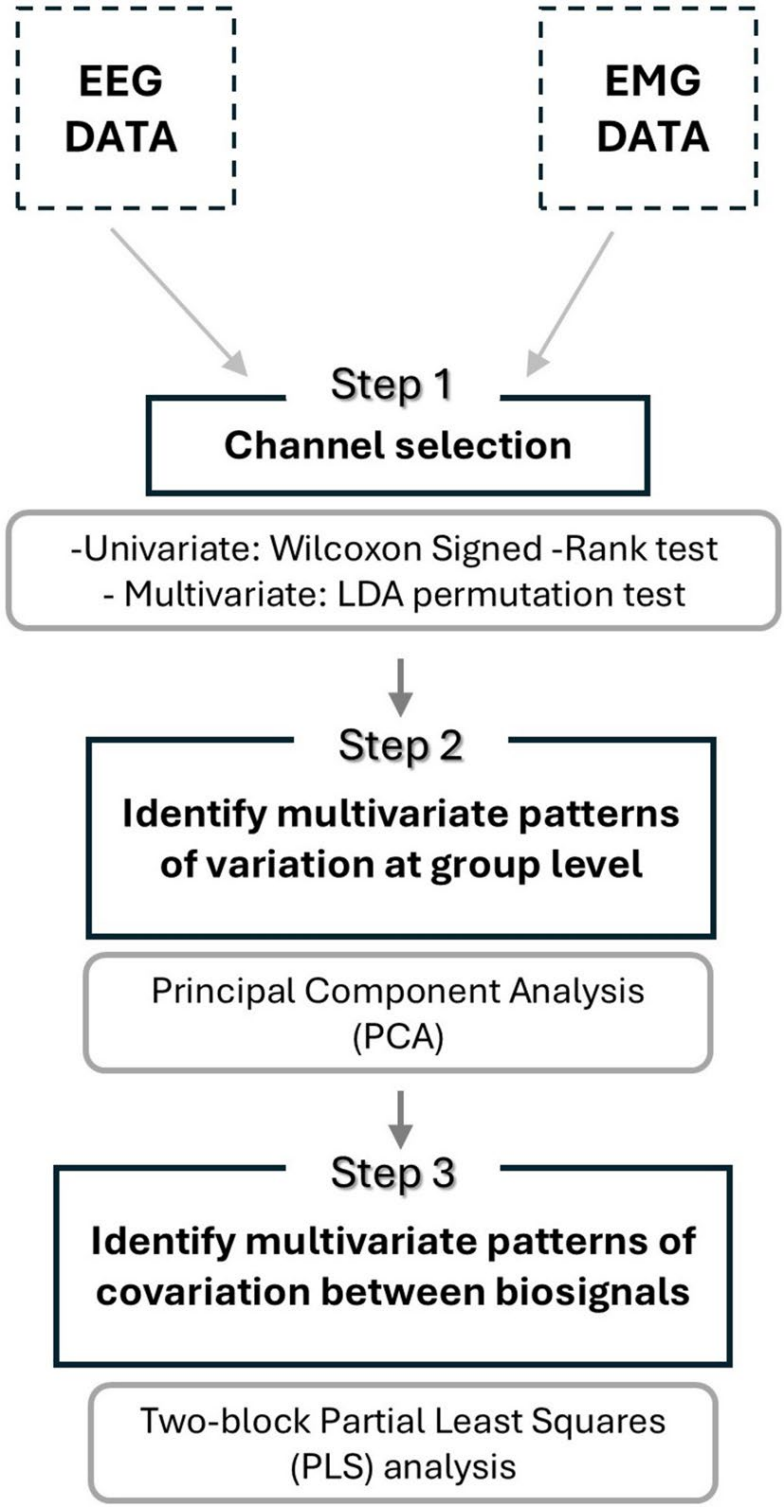
Diagram outlining the key steps of the proposed statistical pipeline for EEG and EMG data analysis

We propose a statistical approach to integrate EEG and EMG data through a multistep procedure aimed at capturing patterns of covariation between neural and muscular activity. The pipeline, schematized in Fig. 1, employs multivariate analysis to explore the interaction between these signals. This approach has been applied to an example dataset reported in the next paragraph. All steps of the analysis were conducted in R (v4.1.0.; R Core Team 2021). Wilcoxon signed-rank test and principal component analysis (PCA) were performed using the ‘stats’ R package (v4.1.0.; R Core Team 2021). Linear discriminant analysis (LDA) was conducted with the ‘MASS’ package (v7.3.54; Venables and Ripley 2002), while the two-block partial least squares (PLS) analysis was performed using the ‘Morpho’ package (v2.9; Schlager 2017). PLS effect scalp maps were generated using ‘eegkit’ (v1.0.4; Helwig 2018).

### Step 1. Channel selection

When recording data using a 32-channel or high-density EEG setup and selecting multiple muscles for EMG measurements, the large number of variables can make it challenging to identify meaningful patterns. In cases where a priori channel selection is not preferred (e.g., exploratory analysis, broad brain region of interest), the first step involves reducing dimensionality by identifying channels that show significant differences or achieve the highest level of discriminatory accuracy between groups. This process ensures that only the most informative variables are retained for subsequent multivariate analyses, enhancing both the efficiency and interpretability of the multimodal investigation.

We propose an approach based on paired comparisons, designed for experimental paradigms aimed at detecting task/condition-related effects. This methodological choice, by directly contrasting two tasks/conditions, offers results that are more intuitive to interpret, as it clearly shows how and to what extent one condition differs from the other. In our study, we aimed to identify EEG and EMG channels that exhibit differences in mean beta power and relative muscle activation between different conditions (i.e., holding the tool and aiming at the tool target) within the same task, and between the same conditions across different tasks (i.e., hammerstone nut-cracking and flake-cutting). Details regarding the experimental design can be found in the next paragraph. EEG channels Fp1, Fp2, TP10, TP9, FT10, and FT9 were excluded a priori from the statistical analysis, as they were observed to be more frequently affected by artifacts in our experiment (refer to Affinito et al. 2024).

After processing the raw data and extracting the relevant features (see the ‘Data acquisition and processing’ section for details on our data preprocessing), we propose two potential approaches for channel selection, referred to as options 1 and 2:

- Option 1: statistical selection via Wilcoxon Signed-Rank Test (Woolson 2005). Apply the Wilcoxon signed-rank test to identify channels exhibiting statistically significant differences between tasks/conditions. Set the desired p-value threshold for statistical significance. For this experiment, an alpha value of 0.05 was used. Rank the significant channels in ascending order based on the magnitude of their Z-scores, following the approach previously employed by Affinito et al. (2024). Select only the channels that meet your predefined criteria. In our case study here, we selected the top five EEG channels and the top five EMG channels for further analysis. When fewer than five channels reached statistical significance, we chose the five channels with the highest absolute Z-scores. This approach ensured consistency in the number of channels analyzed, even in cases where statistical significance was not fully met.
- Option 2: statistical selection via permutation test based on LDA (Good 2000; Tharwat et al. 2017). To assess the discriminatory power of individual channels, perform a permutation test based on LDA for each comparison. For each permutation, five channels are randomly selected, and LDA is applied to calculate the accuracy (%) in discriminating between the two groups. This step ensures a robust assessment of each channel’s contribution to group discrimination. Define the number of permutations to perform. In our case study, the number of permutations was maximized based on the available channel combinations: n = 201,376 for the comparison not involving non-dominant hand muscles, and n = 278,256 for comparisons involving non-dominant hand muscles. This procedure is repeated across all permutations to compute the mean accuracy for each channel. Select only the channels that meet your predefined criteria. In this study, the top five EEG channels and the top five EMG channels are chosen, ranked by their mean accuracy as the most informative for group discrimination.

If the dataset contains a sufficiently large number of channels per modality, it may be useful to perform the permutation test separately for EEG and EMG data. This approach ensures that the most informative channels are independently identified for each modality, preserving their unique contributions during later integration. In this study, both EEG and EMG data are analyzed together to ensure consistency across conditions. The small number of EMG channels—8 channels in total, with only the dominant hand muscles/channels (n = 6) used during the “Hold’” condition (refer to next paragraph)—led to an insufficient number of permutations for a robust analysis. We suggest testing both the separate and combined approaches to assess how each affects the following steps in the analysis pipeline.

It might be informative to compare the outputs (in terms of channels selection and resulting patterns) of both approaches, as each focus on different aspects of the data. The Wilcoxon test identifies which channels show significant differences across conditions on a per-channel basis (univariate test). The LDA-based permutation test considers how channels work together to improve group discrimination (multivariate procedure). In this sense, the results of these two tests can offer complementary perspectives, revealing whether channels that show significant individual effects also contribute meaningfully to group separation in a multivariate context, when interested in variation between groups (i.e., tasks/conditions). If such a comparison is not of interest, the choice between the two approaches can be informed by the nature of the dataset and the specific research question. The univariate approach yields straightforward and easily interpretable results, as it evaluates each channel independently. This method may be more appropriate for smaller datasets and when the aim is to detect strong, localized effects at the channel level, especially when guided by a clear hypothesis or prior knowledge targeting specific channels or well-characterized signal features. The multivariate approach, on the other hand, may be better suited for detecting more distributed patterns that may not be evident when channels are analyzed individually. This can be advantageous when working with larger or complex datasets, where inter-channel interactions are likely of interest. In such cases, this method may highlight channels that do not reach significance on their own but contribute meaningfully when considered in combination. While this step does not capture the full complexity of distributed patterns, it provides a useful foundation for subsequent analyses, such as PLS.

### Step 2. Identify multivariate patterns of variation between groups

The next step involves using PCAs (Jolliffe 2002) to examine the multivariate combination of the selected EEG and EMG channels and identify patterns of variation between the two groups. Perform PCAs based on the correlation matrix and use a scree plot to determine the number of principal components (PCs) to retain when plotting the output. To improve the interpretability of the results, subtract the mean value of the two tasks/conditions from the values of each task/condition for each participant. This approach, previously applied for morphological analyses of bilateral asymmetry (e.g., see Profico et al. 2021), ensures that paired data are centred (mean of 0), making the analysis focus on the relative variation among variables. Given the high inter-individual variability (especially for EEG data) and the fact that each individual is represented twice within the PCA plot (one data point for each task/condition), centering the data facilitates pattern recognition. While the main patterns remain consistent even without this adjustment, centering improves data visualization. This effect can be observed by comparing PC scores before and after mean adjustment using a drop-line graph (e.g., see our previous study by Affinito et al. 2024). It should be noted that, for studies focused on differences across individual participants (e.g., knappers of varying expertise), this centering step is not necessary, and neuromuscular patterns can be compared directly using PCA or PLS, provided that standard statistical recommendations are followed.

Plot the PCA results to visualize group separation and patterns associated with the tasks/conditions. Analyze the loading coefficients of each PC to determine the contribution of each channel to the observed patterns. Focus on channels with high absolute loadings on significant PCs to identify the variables driving group differences. To statistically evaluate differences between the two groups on the PC axes, apply a Wilcoxon signed-rank test to the extracted PC scores, with statistical significance set at p < 0.05. On PC axes where significant differences are found, identify outliers using the interquartile range (IQR) method (Yang et al. 2019). If outliers are detected (1.5 IQR below the first quartile and above the third quartile), evaluate whether to remove them and repeat the analysis (steps 1 and 2) after excluding these participants. Presenting the results before and after the removal of outliers can provide potentially valuable information surrounding the patterns of unusual cases in the dataset and their potential influence on the overall findings. In this way, beyond providing insights into group-specific variation in multivariate patterns, PCA also helps identify potential multivariate outliers that can be excluded from further analyses. For the purposes of this study, we present PCA results based on channels selected using both the Wilcoxon test and the LDA-based permutation test, with the aim of exploring how each selection strategy may influence the resulting multivariate patterns.

### Step 3. identify multivariate patterns of covariation between biosignals

This step employs two-block PLS analysis (Rohlf and Corti 2000) to examine patterns of covariation between EEG and EMG signals. PLS is a multivariate technique that finds linear combinations of the variables in each block (EEG and EMG) that best explain the covariance between the two blocks. This allows for the identification of patterns of covariation between neural and muscular activity, which can provide insight into how the two signals interact during different tasks or conditions.

The two blocks of variables are defined as follows: Block 1 consists of the EEG channels, and Block 2 consists of the EMG channels, both selected using the LDA-based permutation test—channels that have already demonstrated discriminatory accuracy are more likely to reveal meaningful patterns of covariation. If a limited number of EMG channels is present, all available channels may be included, depending on the objectives. For the purpose of this study, which is primarily to demonstrate the basic functionality of the proposed pipeline, all available EMG channels (6 or 8 muscles) were included.

Before performing PLS, it might be beneficial to remove the outliers identified during PCA. Data preprocessing for the PLS involves both centering and scaling of the data. Subtract the mean value of each selected channel (calculated across the two tasks/conditions) from its respective task/condition data. This centers differences between tasks/conditions around zero, facilitating the detection of task/condition-related covariation (see previous step). Scaling standardizes the signals by dividing each variable by its standard deviation. This ensures that EEG and EMG signals are on comparable scales for analysis, allowing the PLS model to focus on the relative relationships between variables rather than their absolute values.

Once the PLS model is computed, plot the scores of the first statistically significant (p < 0.05) PLS component to visualize the covariation between EEG and EMG signals across tasks or conditions. Additionally, the PLS effect (loadings) can be examined to assess the contribution of each variable (EEG and EMG channels) to the covariation.

## Experiment: example dataset on early hominin stone tool use

The study was approved by the Ethics Committee for Psychological Research of the University of Tübingen, in accordance with the international recommendations of the declaration of Helsinki (1964, revised in 2013). The selected tasks represent the earliest documented stone tool use modes in the archaeological record and are fundamental to our understanding of human evolution. For a comprehensive description of the experiment, including a detailed rationale for focusing on these two specific stone tool behaviours and the multi-step trial approach, please refer to our previous publications (Affinito et al. 2024; Eteson et al. 2024b). Below, we provide a summary of the methods and procedures used in this work.

### Subjects

Twenty-three participants (14 females and 9 males), aged 22–55 years (mean age 30.7 ± 7.4 years), from 10 different nationalities and diverse occupational backgrounds, were included in this study. All participants were right-handed, healthy adults with no record of neurological, psychiatric, or musculoskeletal conditions that could compromise task performance. Prior to participation, all subjects provided written informed consent. They attended a session featuring an instructional video that outlined the experimental tasks and provided guidelines for both pre-experiment preparations and conduct during the recording sessions. Immediately before the experiment, participants were given the opportunity to familiarize themselves with the tasks and the manipulation of stone tools.

### Experimental design

The experiment took place at the Max Plank Institute for Intelligent Systems (Tübingen) in a shielded cabin designed to minimize electromagnetic interference. During the data recording, participants performed two tasks, each lasting approximately 30 min. Each task trial was repeated at least 50 times, with a break provided between tasks to prevent fatigue.

Participants engaged in two stone tool-using tasks: one requiring a power grip (percussion task) and another requiring a precision grip (cutting task).

- Task 1: Nut-cracking task. Participants used a replica hammerstone to crack a macadamia nut in a three-step trial: “Hold” (pick up the hammerstone with the dominant hand), “Aim” (place the macadamia nut on the platform with the non-dominant hand and aim at the nut with the tool), “Execute” (strike the nut with the hammerstone).
- Task 2: Cutting task. Participants used a replica Oldowan flake to cut a pre-marked ‘Z’ outline through a faux leather square, following the same three steps as in Task 1: “Hold” (pick up the stone flake with the dominant hand), “Aim” (place the faux leather square on the platform with the non-dominant hand and position the tool along the pre-marked outline), “Execute” (cut through the faux leather square, carefully following the pre-marked ‘Z’ outline).

For all three steps, participants were instructed to maintain the position they had reached after completing the required action, refraining from any further movement until an acoustic cue, presented at 5-s intervals, indicated the transition to the next step.

A control task (i.e., generic finger flexion unrelated to stone tool manipulation) was implemented for EEG measurements, and maximum voluntary contractions (MVC) were recorded to assess each participant’s maximum strength output for specific muscles, using Baseline BIMS power grip and pinch strength dynamometers (Functional model, Fabrication Enterprises, New York).

For the purpose of this study, we will focus on the “Hold” and “Aim” steps of the two tasks to demonstrate the effectiveness of the statistical protocol in integrating EEG and EMG data. The “Execute” was excluded from the pipeline demonstration, as the hammerstone “Execute” condition displayed a persistent artifact resulting from the impact of the hammerstone with the nut, as previously discussed in Affinito et al. (2024).

### Data acquisition and processing

For further details on the EEG/EMG recording and preprocessing pipelines, selection of EEG channels and muscles, as well as the rationale behind these choices, please refer to the protocol by Eteson et al. (2024a, 2025) and our previous studies (Affinito et al. 2024; Eteson et al. 2024b).

EEG and EMG data were recorded simultaneously using a BrainAmp amplifier and BrainVision Recorder software (Version 1.24.0101, Brain Products GmbH, Gilching, Germany) at a sampling rate of 2500 Hz. EEG data were acquired from 32 electrodes placed on the scalp according to the international 10–20 system, with the central frontal channel (FCz) serving as the online reference. EMG data were collected from 8 muscles (or muscle groups) using surface sensors applied to the skin of the upper limb, in accordance with the SENIAM guidelines (Stegeman and Hermens 2007). The selected muscles included the *flexor carpi radialis* (FCR), *flexor carpi ulnaris* (FCU), *flexor pollicis longus* (FPL), the thenar eminence muscle group (TE) consisting of *abductor pollicis brevis*, *flexor pollicis brevis*, and *opponens pollicis*, the first dorsal *interosseus* (DI1), and the hypothenar eminence muscle group (HTE), which includes *abductor digiti minimi*, *flexor digiti minimi*, and *opponens digiti minimi*. Additionally, EMG measurements for DI1 and the TE group were recorded from the non-dominant hand.

Data preprocessing was conducted separately for EEG and EMG using BrainVision Analyzer software (Version 2.2, Brain Products GmbH, Gilching, Germany). For EEG data, signals were downsampled to 250 Hz and filtered using an IIR filter with a low cutoff of 1 Hz, a high cutoff of 40 Hz, and a 50 Hz notch filter. Data were re-referenced using the reference electrode standardization technique (REST) (Yao 2001). Ocular artifacts and persistent muscle artifacts were removed using independent component analysis (ICA) based on the Infomax algorithm (Bell and Sejnowski 1995; Lee et al. 1999). Baseline correction was applied to the entire trial, after which each step (e.g., “Aim”) was epoched from 0 to 1000 ms. Segments (repetitions of the same step) containing residual artifacts were excluded. To extract mean beta activity (µV^2^) for each step of the trial, a Fast Fourier Transformation (FFT) with a 10% Hanning window was performed on the remaining segments. These segments were then averaged to compute the mean power spectrum for each task step per participant.

For EMG data, signals from each of the 8 muscles were downsampled to 500 Hz and filtered using an IIR filter (low cutoff 20 Hz, high cutoff 450 Hz). Data were then rectified, and each trial step was segmented from 0 to 4000 ms. These segments, representing repetitions of the same step, were averaged and exported in 0.25-s intervals. The exported values were further averaged to produce a single value per step for each participant. Maximum voluntary contraction (MVC) data were used to calculate individual percentage maximum voluntary contractions (%MVC) which served as the basis for assessing relative muscle activation during each task step. This normalization enabled the comparison of muscle activation across participants.

## Results

In the following sections, we present the results from applying each step of the statistical pipeline to an example dataset. The dataset originates from an experiment investigating early hominin stone tool use, structured around two distinct tasks: hammerstone nut-cracking and flake cutting. Each task consists of three distinct steps within the experimental procedure. For this study, we focus on the “Hold” and “Aim”, reporting differences both within each type of condition and between the two different conditions within the same task.

## Channel selection and outliers

The first step of the proposed pipeline applies a statistical approach to select a subset of EEG and EMG channels. For illustrative purposes, we present the results from our dataset using both selection strategies: option 1, which selects channels based on statistically significant differences, and option 2, which selects channels with the highest discriminatory accuracy across tasks/conditions.

The Wilcoxon signed-rank test (option 1) is used to identify significant differences in EEG beta power and EMG activation. The results for all comparisons, including data from all participants, are presented in Table 1. Significant differences in EEG and EMG channels are observed across all comparisons. Specific findings include:

- Nut-cracking Hold vs. cutting Hold: five EEG channels, predominantly located at contralateral frontoparietal regions, show significant differences in beta power (p < 0.05). All six EMG channels show significant differences in muscle activation (p < 0.001).
- Nut-cracking Aim vs. cutting Aim: only two EEG channels, located at posterior parietal and occipital regions on the right hemisphere, exhibit significant differences in beta power. All EMG channels except the non-dominant DI1 muscle show significant differences (p < 0.001).
- Hold vs. Aim (flake use): twelve EEG channels are identified, spanning bilateral frontoparietal, left temporal, and bilateral motor cortical regions. Significant differences are also observed in six EMG channels, including non-dominant hand muscles (p < 0.001) and excluding the FPL and HTE muscles.
- Hold vs. Aim (hammerstone use): fourteen EEG channels, predominantly located at left frontoparietal and temporal, as well as bilateral motor cortex regions, display significant differences. Additionally, four EMG channels exhibit significant differences including the non-dominant hand muscles (p < 0.001).

**Table 1:** Wilcoxon signed-rank test results for the four paired comparisons, including data from all 23 participants

Channels	H-Hold vs F-Hold		F-Hold vs F-Aim		H-Hold vs H-Aim		H-Aim vs F-Aim	
	Z scores	p value	Z scores	p-value	Z scores	p value	Z scores	p value
Fz	−1,5360	0.1245	−0.4714	0.6373	−3.4825	0.0005***	−0.0760	0.9394
F3	−1.9618	0.0498*	−3.4521	0.0006***	−4.0908	4.30E−05***	−1.9009	0.0573
F7	−0.3194	0.7495	−2.1747	0.0297*	−2.4180	0.0156*	−0.1673	0.8671
FC5	−1.2926	0.1961	−3.6650	0.0002***	−2.9959	0.0027**	−1.3535	0.1759
FC1	−2.3572	0.0184*	−1.8401	0.0658	−3.5129	0.0004***	−0.0760	0.9394
C3	−0.0152	0.9879	−3.0567	0.0022**	−3.3000	0.0010***	−0.4106	0.6814
T7	−0.6235	0.5330	−3.7867	0.0002***	−2.3572	0.0184*	−0.4106	0.6814
CP5	−1.0493	0.2940	−3.2088	0.0013**	−3.3000	0.0010***	−0.6843	0.4938
CP1	−2.4180	0.0156*	−1.2014	0.2296	−2.9046	0.0037**	−0.1065	0.9152
Pz	−1.6576	0.0974	−0.6235	0.5330	−1.1406	0.2541	−0.9277	0.3536
P3	−0.1673	0.8671	−1.5968	0.1103	−2.5396	0.0111*	−0.8972	0.3696
P7	−0.1673	0.8671	−1.9313	0.0534	−2.7526	0.0059**	−0.3802	0.7038
O1	−0.9581	0.3380	−0.3802	0.7038	−0.5931	0.5531	−1.1101	0.2669
Oz	−1.8401	0.0658	−0.3802	0.7038	−0.6235	0.5330	−1.9009	0.0573
O2	−2.4788	0.0132*	−0.7756	0.4380	−0.0760	0.9394	−2.7830	0.0054**
P4	−1.3231	0.1858	−2.0530	0.0401*	−0.8060	0.4202	−1.8097	0.0703
P8	−1.9313	0.0534	−1.3231	0.1858	−0.6843	0.4938	−2.3572	0.0184*
CP6	−1.8097	0.0703	−2.1747	0.0297*	−1.9618	0.0498*	−1.1406	0.2541
CP2	−1.5360	0.1245	−2.2659	0.0235*	−0.0152	0.9879	−0.6843	0.4938
Cz	−2.2963	0.0217*	−1.4447	0.1485	−2.2051	0.0274*	−0.2889	0.7726
C4	−0.0152	0.9879	−3.7867	0.0002***	−2.0834	0.0372*	−1.3535	0.1759
T8	−0.1369	0.8911	−1.2926	0.1961	−0.3498	0.7265	−0.0152	0.9879
FC6	−0.9277	0.3536	−1.1710	0.2416	−0.5627	0.5737	−0.4106	0.6814
FC2	−1.7489	0.0803	−1.9618	0.0498*	−1.4447	0.1485	−0.3498	0.7265
F4	−1.5055	0.1322	−0.4714	0.6373	−0.4106	0.6814	−0.8364	0.4029
F8	−0.7756	0.4380	−2.5396	0.0111*	−0.8364	0.4029	−0.2585	0.7960
ndTE	–	–	−4.1821	2.89E−05***	−4.1821	2.89E−05***	−2.0834	0.0372*
FCU	−4.1821	2.89E−05***	−2.1747	0.0297*	−0.6843	0.4938	−4.0908	4.30E−05***
FCR	−4.1821	2.89E−05***	−3.2696	0.0011**	−1.9618	0.0498*	−4.1821	2.89E−05***
FPL	−4.1821	2.89E−05***	−0.3802	0.7038	−0.1065	0.9152	−4.1516	3.30E−05***
TE	−2.0530	0.0401*	−2.5092	0.0121*	−0.0456	0.9636	−3.3609	0.0008***
DI1	−3.5433	0.0004***	−2.5092	0.0121*	−2.2659	0.0235*	−3.3000	0.0010***
HTE	−4.1516	3.30E−05***	−1.4751	0.1402	−1.1710	0.2416	−4.1516	3.30E−05***
ndDI1	**–**	**–**	−4.1821	2.89E−05***	−4.1821	2.89E−05***	−1.3535	0.1759

Fz-F8 channels correspond to EEG electrodes following the 10–20 international system (32-channel configuration)

Significant p-values are highlighted in bold

*FCR* flexor carpi radialis, *FCU* flexor carpi ulnaris, *FPL* flexor pollicis longus, *TE* thenar eminence muscle group, *HTE* hypothenar eminence muscle group, *DI1* first dorsal interosseous, *ndDI1* non-dominant first dorsal interosseous, *ndTE* non-dominant thenar eminence group

p < 0.001*** p < 0.01** p < 0.05*

The second statistical approach (option 2) relies on a permutation test based on LDA to evaluate the accuracy of EEG and EMG channels in distinguishing between the two groups compared in each case. Results for all comparisons, including data from all participants, are detailed in Table 2, and reveal an overall pattern consistent with those observed in the Wilcoxon test:

- Within-condition comparisons (Hold vs. Hold; Aim vs. Aim): channels from muscle EMG components—specifically FCR, HTE, FCU, and FPL—are the first to show increased discriminatory accuracy, achieving approximately 80% accuracy.
- Between-condition comparisons (Hold vs. Aim): for both flake and hammerstone use, high discriminatory accuracy is primarily associated with EMG channels corresponding to muscles of the non-dominant hand, as well as EEG channels covering the motor cortex and frontoparietal regions.

**Table 2:** LDA-based permutation test results for the four paired comparisons, including data from all 23 participants

n = 201,376 permutations		n = 278,256 permutations		n = 278,256 permutations		n = 278,256 permutations	
H-Hold vs F-Hold		F-Hold vs F-Aim		H-Hold vs H-Aim		H-Aim vs F-Aim	
Channels	Accuracy (%)	Channels	Accuracy (%)	Channels	Accuracy (%)	Channels	Accuracy (%)
FCR	82.2653	ndTE	94.0534	ndTE	88.9647	FCR	88.7649
HTE	80.3416	ndDI1	90.5349	ndDI1	73.8249	FPL	81.2571
FCU	79.7241	C3	72.6244	C4	67.3289	HTE	81.2567
FPL	79.1279	C4	71.6583	FC5	67.1350	FCU	81.1890
O2	74.6976	CP1	70.4067	FC1	67.0320	TE	77.1712
DI1	73.0502	FC1	70.0203	F3	66.3755	O2	77.1350
Oz	72.7266	F3	70.0143	P7	66.2374	ndTE	75.7556
TE	72.3218	TE	69.9050	C3	66.1059	Oz	74.2739
CP6	72.2677	FC2	69.8793	CP1	65.6831	DI1	74.1549
CP5	72.1209	CP2	69.7986	Fz	65.5734	P8	74.0476
P4	72.0837	P4	69.6644	CP5	65.2262	P4	72.8557
F3	71.9165	FC5	69.5278	TE	65.1377	CP6	72.7649
Cz	71.7932	Fz	69.2363	FC2	65.1334	ndDI1	72.4809
P8	71.7238	CP5	69.1848	FC6	65.1108	CP5	72.3443
FC1	71.6273	DI1	69.1778	T7	65.0232	CP1	72.1092
CP1	71.3628	FCU	69.0726	DI1	65.0060	F3	71.9832
T8	71.0954	Cz	69.0459	CP2	64.7726	Pz	71.7860
C3	70.9933	T7	68.9046	FCR	64.7216	C3	71.7767
FC2	70.9701	FCR	68.8783	F7	64.6409	F4	71.7374
Fz	70.9355	P8	68.7666	P4	64.5456	CP2	71.7324
P3	70.9353	Pz	68.6397	P8	64.5220	FC5	71.6907
P7	70.8885	CP6	68.5448	F4	64.5017	Cz	71.6837
O1	70.8319	T8	68.4941	T8	64.5009	Fz	71.6153
FC6	70.8266	FPL	68.4393	CP6	64.4668	O1	71.5914
T7	70.7333	P3	68.4377	P3	64.4122	C4	71.5913
CP2	70.7210	O2	68.3998	HTE	64.3408	T8	71.5868
F4	70.7207	F7	68.3758	Cz	64.3099	FC2	71.4315
F8	70.7118	F4	68.3505	Pz	64.2943	P3	71.4308
Pz	70.4800	HTE	68.3103	FPL	64.2514	FC1	71.3909
FC5	70.4551	F8	68.2957	O1	64.2377	T7	71.3202
C4	70.4207	Oz	68.2607	O2	64.2325	FC6	71.3196
F7	70.2895	O1	68.0832	FCU	64.1930	F7	71.3023
		P7	68.0825	Oz	64.1259	F8	71.2642
		FC6	67.9320	F8	64.1147	P7	71.1036

*FCR* flexor carpi radialis, *FCU* flexor carpi ulnaris, *FPL* flexor pollicis longus, *TE* thenar eminence muscle group, *HTE* hypothenar eminence muscle group, *DI1* first dorsal interosseous, *ndDI1* non-dominant first dorsal interosseous, *ndTE* non-dominant thenar eminence group

All other channels correspond to EEG electrodes following the 10–20 international system (32-channel configuration)

To demonstrate the pipeline, we select the top five EEG and five EMG channels using both option 1 and option 2, with each set of selected channels processed separately in the subsequent step of the pipeline (i.e., PCA). As mentioned in materials and methods, the PCA also allows the identification of potential multivariate outliers in the dataset. For comparisons where outliers were detected, the two-channel selection processes (tests) were repeated after removing the outliers. In either case, the results, presented in Supplementary Tables S1, and S2, show that removing outliers had minimal impact on the overall outcomes in our dataset. No major differences are observed in the channels showing significant differences or in the accuracy of discrimination, and the final selection of the top 10 channels (5 EEG and 5 EMG) remains largely consistent before and after outlier removal (compare Table 1 with Supplementary Table S1 and Table 2 with Supplementary Table S2). The only differences observed after removing the outliers are as follows:

- Wilcoxon test: in the paired comparison of the nut-cracking Aim vs. cutting Aim condition, EEG channel P4 is replaced in the top five list by channel C4. However, neither P4 nor C4 exhibits statistically significant differences in either case.
- Permutation test: in the nut-cracking Hold vs. cutting Hold comparison, EEG channel P4 is replaced in the top five list by channel F3. However, P4 and F3 have nearly identical discriminatory accuracy values. In the Hold vs. Aim comparison for hammerstone use, the muscle TE is replaced in the top five list by FPL, again with both muscles showing very similar accuracy values.

In the present demonstration dataset, we chose to remove outliers to show their potential impact. The observed differences in selected channels following outlier removal are primarily attributable to the stringent criterion employed, which limited selection to only the top five channels per modality. Consequently, when multiple channels exhibit comparable scores, the exclusion of an outlier may alter the channel ranking and lead to a different subset of selected channels, which might have implications for the different brain regions considered. This effect could be mitigated by adopting a less restrictive selection criterion. Nonetheless, we recommend evaluating the presence of multivariate outliers in the dataset and making decisions on their removal on a case-by-case basis, taking into account the specific research objectives. For instance, outliers reflecting isolated noise resulting in artificially high values should generally be excluded, whereas outliers reflecting higher values yet consistent with the overall data pattern may not necessitate removal.

It is interesting to note that difference between the two selection methods emerges when comparing results across EEG and EMG channels. The 5 EMG channels selected are nearly identical across methods, suggesting consistency in the identification of informative muscle components. In contrast, the selection of the five EEG channels shows differences between the Wilcoxon test and the LDA-based permutation test, with the majority of EEG channels differing between the two methods (Table 3). The EEG channels that were consistently identified in both tests are F3, FC1, C4, O2, and P8. This discrepancy could likely reflect differences in the nature and characteristics of the two signals. EMG activity is typically more localized and directly linked to specific muscles, resulting in a relatively simple and stable signal structure that is consistently captured by both univariate and multivariate analyses. EEG, on the other hand, captures more complex and spatially distributed neural dynamics which can result in different subsets being highlighted depending on whether the analysis emphasizes individual channel effects or multivariate interactions.

**Table 3:** Selection of 10 channels (5 EEG, 5 EMG) based on Wilcoxon signed-rank test and LDA-based permutation test, excluding PCA outliers

	H-Hold vs F-Hold		F-Hold vs F-Aim		H-Hold vs H-Aim		H-Aim vs F-Aim	
	EEG	EMG	EEG	EMG	EEG	EMG	EEG	EMG
Wilcoxon signed-rank test	O2*	HTE***	T7***	ndTE***	F3***	ndTE***	O2**	FCU***
	CP1*	FCR***	F3***	ndDI1***	FC1***	ndDI1***	P8*	FCR***
	FC1*	FCU***	FC5***	FCR**	C3**	DI1*	Oz	FPL***
	Cz*	FPL***	C4***	TE*	CP5**	FCR	F3	HTE***
	F3*	DI1***	CP5**	DI1*	Fz***	HTE	C4	TE**
LDA-based permutation test	O2 (75%)	FCR (82%)	C3 (73%)	ndTE (94%)	C4 (69%)	ndTE (94%)	O2 (76%)	FCR (89%)
	Oz (74%)	HTE (80%)	C4 (72%)	ndDI1 (91%)	FC1 (69%)	ndDI1 (76%)	P8 (75%)	HTE (84%)
	CP5 (73%)	FCU (79%)	CP1 (70%)	TE (70%)	P7 (69%)	FCR (67%)	Oz (75%)	FCU (82%)
	CP6 (72%)	FPL (78%)	FC1 (70%)	DI1 (69%)	F3 (68%)	DI1 (67%)	P4 (74%)	FPL (82%)
	F3 (72%)	DI1 (73%)	F3 (70%)	FCU (69%)	FC5 (68%)	FPL (66%)	CP6 (73%)	TE (77%)

Accuracy values are provided in parentheses

*FCR* flexor carpi radialis, *FCU* flexor carpi ulnaris, *FPL* flexor pollicis longus, *TE* thenar eminence muscle group, *HTE* hypothenar eminence muscle group, *DI1* first dorsal interosseous, *ndDI1* non-dominant first dorsal interosseous, *ndTE* non-dominant thenar eminence group

All other channels correspond to EEG electrodes following the 10–20 international system (32-channel configuration)

p < 0.001*** p < 0.01** p < 0.05*

## Patterns of variation between groups

**Fig. 2:**
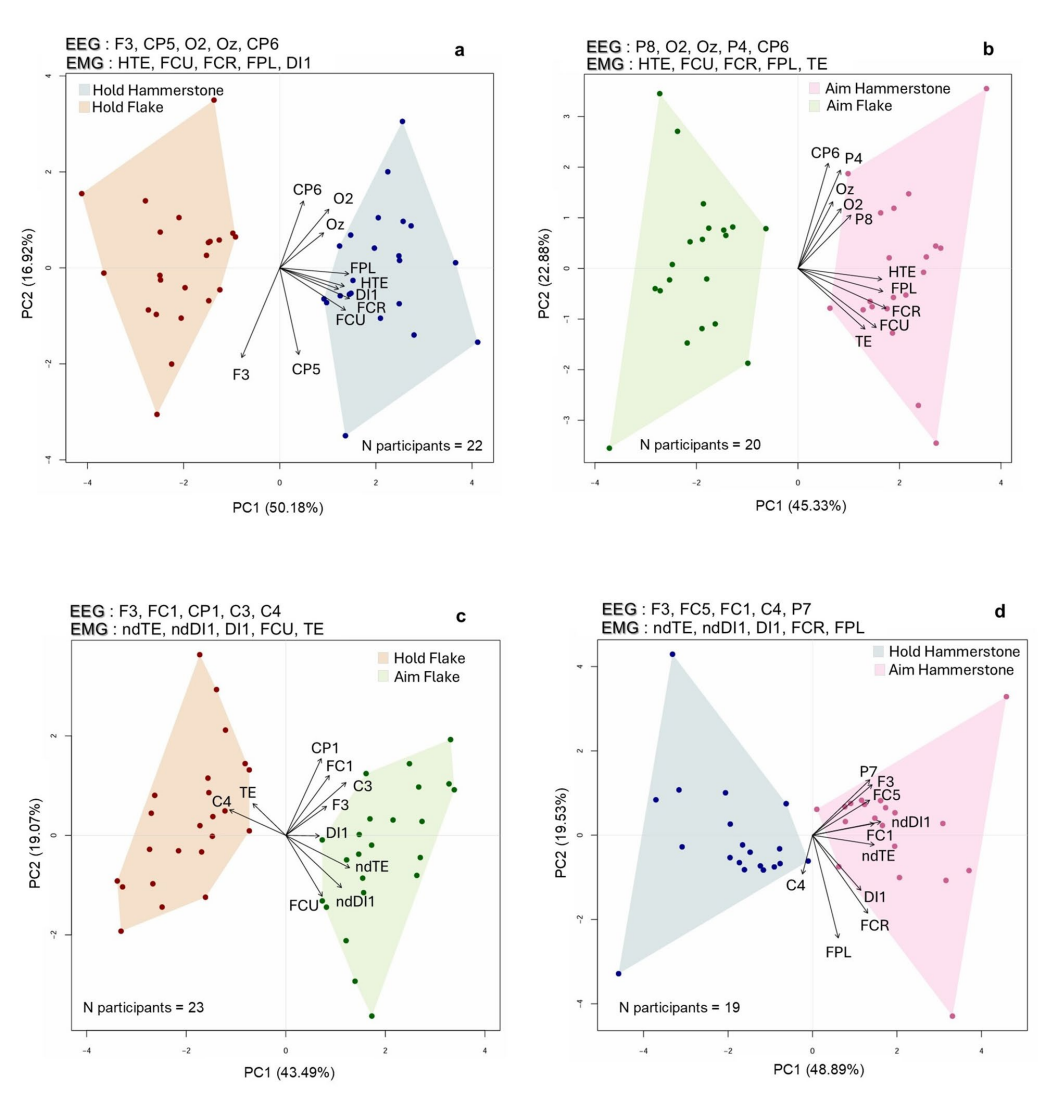
Principal component analysis (PCA): comparisons within the same experimental condition—Hold vs. Hold (**a**) and Aim vs. Aim (**b**) in both flake and hammerstone use—as well as between different conditions—Hold vs. Aim for flake use (**c**) and hammerstone use (**d**). The analysis was performed using the 10 EEG and EMG channels selected based on the highest accuracy values in the LDA-based permutation test selection approach. The plots reflect PCA results after outlier removal. *FCR* flexor carpi radialis, *FCU* flexor carpi ulnaris, *FPL* flexor pollicis longus, *TE* thenar eminence muscle group, *HTE* hypothenar eminence muscle group, *DI1* first dorsal interosseous, *ndDI1* non-dominant first dorsal interosseous, *ndTE* non-dominant thenar eminence group. All other channels correspond to EEG electrodes following the 10–20 international system (32-channel configuration)

The PCA results for the channels selected using the LDA-based permutation test are presented in Fig. 2, while those from the channels selected using the Wilcoxon test are shown in Supplementary Figure S1. Below, we describe the resulting patterns observed for each selection strategy. Since the LDA-based permutation approach was also used as the basis for PLS analysis (refer to materials and methods), we present the corresponding PCA results first.

### Permutation-based channel selection

For the comparison between holding a hammerstone and holding a flake (Fig. 2a), the first two PCs collectively account for 67.1% of the variance (PC1 = 50.18%; PC2 = 16.92). A clear separation between the two experimental steps is observed along PC1, where the variations in PC values are also statistically significant (p < 0.001—refer to Supplementary Table S3). In contrast, no significant variation is detected along PC2. Hammerstone holding is associated with positive PC1 values, while flake holding corresponds to negative PC1 values. The loading values, reported in Supplementary Table S3, indicate that all muscle variables have high positive loadings on PC1. This reflects relatively higher muscle activation during hammerstone holding, which represents the dominant aspect of the observed variation in this comparison. While the EEG channels are not the primary drivers of variation along PC1, occipital channels also show positive loadings, and the F3 channel is the only one showing a negative loading value, suggesting a proportional increase in beta power during flake holding.

For the comparison between aiming with a hammerstone and aiming with a flake (Fig. 2b), the first two PCs collectively account for 68.2% of the variance (PC1 = 45.33%; 22.88%). A clear separation between the two experimental conditions is observed along PC1, with statistically significant variations (p < 0.001—refer to Supplementary Table S4). No significant variation is observed along PC2. Hammerstone aiming is located at positive PC1 values, while flake aiming is located at negative PC1 values. As in the previous comparison, all muscle variables have high positive loadings on PC1 (see Supplementary Table S4), driving the majority of the variation. EEG channels, including O2 and parietal channels on the right hemisphere, also contribute to the separation between conditions. Although their contribution is not as dominant as the muscles, it indicates relatively increased beta power in these channels during hammerstone aiming.

For the comparison between holding and aiming with a flake (Fig. 2c), the first two PCs account for 62.6% of the variance (PC1 = 43.49; PC2 = 19.07). A clear separation between the two compared conditions is observed along PC1 (p < 0.001—refer to Supplementary Table S5), while no separation or significant variation is observed along PC2. Holding is associated with negative PC1 values, while aiming corresponds to positive PC1 values. The loadings, reported in Supplementary Table S5, show that the most dominant aspect of this variation is: the high positive loadings of variables corresponding to non-dominant hand muscles and C3, indicating relatively increased muscle activation and beta power in these channels during aiming; the high negative loading of C4, reflecting relatively higher beta power during holding compared to aiming. Although less pronounced, frontoparietal channels included in this comparison are positively loaded, suggesting proportionally higher beta power during aiming.

For the comparison between holding and aiming with a hammerstone (Fig. 2d), the first two PCs collectively account for 68.4% of the variance (PC1 = 48.89%; PC2 = 19.53%). A clear separation is observed along PC1 (p < 0.001—refer to Supplementary Table S6). Holding is associated with negative PC1 values, while aiming corresponds to positive PC1 values. The loadings, reported in Supplementary Table S6, indicate that the dominant aspect of variation is the high positive loadings of variables representing non-dominant hand muscles and frontoparietal EEG channels. This reflects relatively increased muscle activation and beta power in these regions while aiming.

### Wilcoxon-based channel selection

Regarding the patterns observed in the Wilcoxon test channel selection (Supplementary Figure S1), the overall results are broadly consistent with those from the permutation test approach, despite the aforementioned differences in the selected EEG channels. On average, the first two principal components (PCs) account for approximately 65% of the variance, with PC1 consistently exhibiting a clear and statistically significant separation between the compared conditions (refer to Supplementary Tables S7, S8, S9, and S10).

For comparisons between the two tasks (nut-cracking and cutting) for the same condition (Hold vs. Hold and Aim vs. Aim), the primary source of variation is consistently linked to the levels of muscle activity, with relatively greater muscle activation observed in hammerstone use (see Supplementary Tables S7 and S8 for loading values). Focusing on the EEG channels, in the Hold vs. Hold comparison (Supplementary Figure S1a), frontoparietal channels have positive loadings on PC1, indicating relatively higher beta power when holding a flake. In contrast, in the Aim vs. Aim comparison (Supplementary Figure S1b), while the separation along PC1 remains primarily driven by muscle activity, distinct EEG contributions emerge: occipital and parietal channels have positive loadings, while F3 has a negative loading value. This pattern suggests higher beta power for hammerstone use in the occipital and parietal regions and higher beta power for flake use in F3. However, F3 does not show a statistically significant difference in the Wilcoxon test but was included based on our predefined selection criteria (refer to Materials and Methods), which ensured that five channels were selected consistently for each comparison.

For comparisons between different conditions within each task (i.e., Hold vs. aim in both flake use and hammerstone use—Supplementary Figures S1c and d), the separation along PC1 is primarily influenced by non-dominant hand muscle activity, motor cortex channels, and frontoparietal channels (refer to Supplementary Tables S9 and S10 for loading values), following a similar trend to the permutation-based selection PCA. Specifically, all non-dominant hand muscles and frontoparietal channels show relative increase during aiming, regardless of whether a flake or a hammerstone is used.

## Patterns of covariation between EEG and EMG

**Fig. 3:**
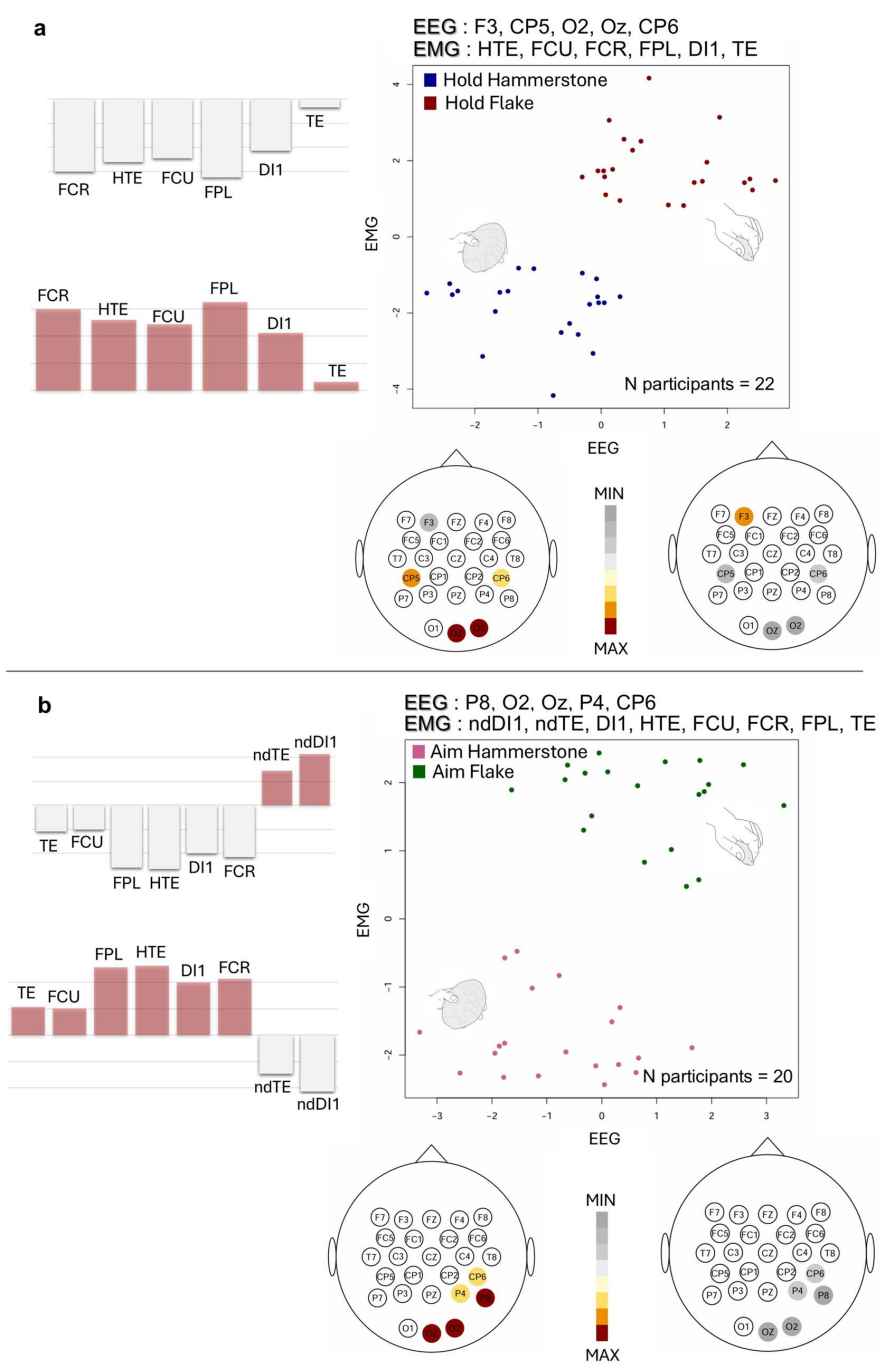
Two-block partial least squares (PLS) analysis: plots of the first PLS axis reporting the covariation between EEG and EMG blocks when comparing the same experimental condition—Hold vs. Hold (**a**) and Aim vs. Aim (**b**) for both flake and hammerstone use. The analysis was performed using the 5 EEG channels selected based on the highest accuracy in the LDA-based permutation test and all EMG channels, after PCA outliers’ removal. PLS loadings relative to the EEG block are represented as scalp maps below each plot, while the PLS loadings relative to the EMG block are shown to the side of each plot. *FCR* flexor carpi radialis, *FCU* flexor carpi ulnaris, *FPL* flexor pollicis longus, *TE* thenar eminence muscle group, *HTE* hypothenar eminence muscle group, *DI1* first dorsal interosseous, *ndDI1* non-dominant first dorsal interosseous, *ndTE* non-dominant thenar eminence group. All other channels correspond to EEG electrodes following the 10–20 international system (32-channel configuration)

**Fig. 4:**
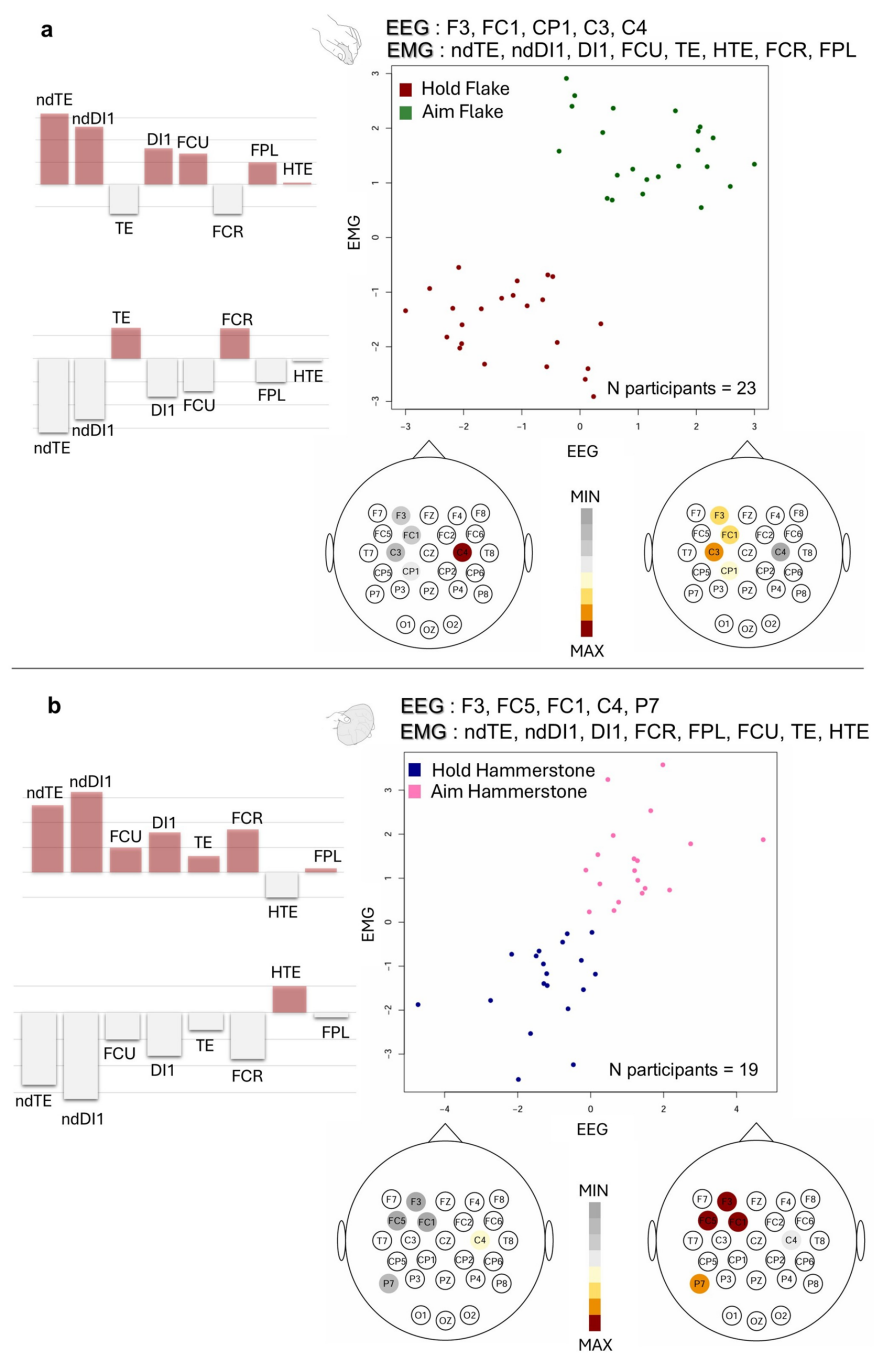
Two-block partial least squares (PLS) analysis: plots of the first PLS axis reporting the covariation between EEG and EMG blocks when comparing different conditions within the same task—Hold vs. Aim for flake use (**a**) and hammerstone use (**b**). The analysis was performed using the 5 EEG channels selected based on the highest accuracy in the LDA-based permutation test and all EMG channels, after PCA outliers’ removal. PLS loadings relative to the EEG block are represented as scalp maps below each plot, while the PLS loadings relative to the EMG block are shown to the side of each plot. *FCR *flexor carpi radialis, *FCU* flexor carpi ulnaris, *FPL* flexor pollicis longus, *TE* thenar eminence muscle group, *HTE* hypothenar eminence muscle group, *DI1* first dorsal interosseous, *ndDI1* non-dominant first dorsal interosseous, *ndTE* non-dominant thenar eminence group. All other channels correspond to EEG electrodes following the 10–20 international system (32-channel configuration)

To examine the covariation between EEG (block1) and EMG (block2) across tasks or conditions, a two-block PLS analysis was performed. The results of the PLS for all the comparisons are presented in Fig. 3 and Fig. 4.

Nut-cracking Hold vs. cutting Hold: the two-block PLS analysis indicates a significant correlation between the first PLS axes of each block (r = 0.62, p = 0.001—see Fig. 3a and Supplementary Table S11), explaining 93% of the total covariance between EEG and EMG. At negative PLS axis values, relatively high activation of all selected muscles—particularly FPL and FCR, with minimal TE involvement—is observed. This pattern co-occurs with relatively increased activity in the ipsilateral occipital and CP5 channels, while F3 activation remains proportionally low (i.e., pattern predominantly observed in Hold hammerstone). In contrast, positive PLS axis values are associated with relatively high F3 activation that coincides with proportionally reduced activity across all selected muscles (i.e., pattern predominantly observed in Hold flake).

Nut-cracking Aim vs. cutting Aim: the two-block PLS analysis reveals a significant correlation among the first PLS axes of EEG and EMG (r = 0.49, p = 0.028—Fig. 3b and Supplementary Table S12), capturing 82% of the total covariance between blocks. At negative values of the PLS axes, high activation of all selected muscles—particularly FPL, HTE and FCR—except the non-dominant hand muscles, are associated with relatively high activity in the right occipital and parietal EEG channels (i.e., the pattern predominantly observed in Aim with a hammerstone). In contrast, at positive PLS axis values, increased involvement of non-dominant hand muscles shows a negative correlation with activity in all EEG channels (i.e., patterns predominantly observed in Aim with a flake).

Hold vs. Aim in flake use: the two-block PLS analysis indicates a significant correlation among the first PLS axes of each block (r = 0.64, p = 0.001—Fig. 4a and Supplementary Table S13), capturing 85% of the total covariance between EEG and EMG. Positive PLS axis values are associated with high non-dominant hand muscles activity—along with, to a lesser extent, DI1, FCU and FPL. This pattern co-occurs with high activity in C3 and frontal channels and low activity in C4 channel (pattern predominantly observed in Aim). At negative values of the PLS axes, relatively high C4 activity and low C3 and frontal channels activity co-occur with proportionally reduced non-dominant hand muscle activity and increased TE and FCR activation (i.e., the pattern predominantly observed in Hold).

Hold vs. Aim in hammerstone use: the two-block PLS analysis indicates a significant correlation among the first PLS axes of EEG and EMG (r = 0.72, p = 0.001—Fig. 4b and Supplementary Table S14), explaining 93% of the total covariance between blocks. Positive PLS axis values are associated with relatively high activity in non-dominant hand muscles—along with, to a lesser extent, FCU, DI1, TE, and FCR, with proportionally minimal FPL involvement. This pattern co-occurs with relatively high activity in frontoparietal channels and low activity in C4 (i.e., the pattern predominantly observed in Aim).

## Discussion

This study introduces a novel statistical pipeline for integrating and analysing EEG and EMG data in experimental neuroarchaeology, with the Aim of investigating brain-hand interactions during stone tool-related behaviours. By combining a range of multivariate techniques, this multistep approach aims to uncover meaningful dynamics between EEG and EMG signals by (1) identifying the most relevant channels in group-level comparisons, (2) characterizing patterns of variation across different tasks or conditions, and (3) identifying patterns of covariations between neural and muscular activity. We demonstrate the utility of this pipeline using data from a previously conducted experiment on early hominin stone tool use, in which participants performed a hammerstone nut-cracking task and a flake-cutting task (Affinito et al. 2024; Eteson et al. 2024b). For the purpose of this study, we focused on the “Hold” and “Aim” task conditions, during which participants picked up the tool and then aimed at the task target (a nut or a pleather square).

Overall, our analyses highlight the efficacy of the integrated approach in capturing independent and combined contributions of neural and muscular signals during manual tasks. Through our proposed multistep approach, we identified clear differences between conditions and found significant EEG–EMG covariations. The selected channels effectively differentiated the two tool-using tasks under investigation. Within-condition comparisons revealed that muscle activity played a predominant role in distinguishing task-specific variations. Hammerstone use was associated with proportionally increased muscle activation, likely reflecting the heavier weight of the tool and the need for a stable grip. In contrast, the flake cutting task exhibited relatively increased beta activation in the frontal area, consistent with our previous findings (Affinito et al. 2024) and the proposed hypotheses surrounding the cognitive implications of precise manipulation in hominins. The introduction of cutting tools, typically associated with the Oldowan industry (Semaw et al. 2003; Braun et al. 2019), is regarded as a significant development in hominin behavior. While percussive activities like nut-cracking are well-documented among some non-human primates, the intentional production and use of sharp-edged tools appear to be characteristic of hominins. This transition naturally offered certain adaptive advantages, with this behaviour often interpreted as indicative of emerging cognitive and motor skills, although the exact extent and nature of this remain subjects of ongoing research (e.g., Stout and Chaminade 2007; Braun et al. 2019; Bryche et al. 2024).

When examining differences within each task (i.e., across conditions), the “Aim” condition exhibited a positive covariation between non-dominant hand muscle activity and frontoparietal EEG channels (refer to Fig. 4), indicating enhanced bimanual coordination and cortical involvement. Conversely, a negative covariation was observed between non-dominant hand muscles and the ipsilateral motor cortex, likely reflecting event-related desynchronization (ERD) in the beta band over C4 (Crone 1998; Zaepffel et al. 2013), as the non-dominant hand also contributed to the aiming movement (see Fig. 4; for details, refer to Affinito et al. 2024). This pattern was particularly pronounced in the hammerstone condition (Fig. 4b), which, in the PLS analysis of Hold vs. Aim, showed the strongest EEG–EMG correlation (r = 0.72). This suggests a more graded increase in frontoparietal beta activity and bimanual muscular engagement, likely reflecting the elevated motor and cognitive demands involved in planning and executing a percussive action. In contrast, the flake condition (Fig. 4a) displayed a broader distribution of axis scores in the PLS comparison between Hold and Aim, potentially indicating greater inter-individual variability in precision grip strategies, which may give rise to more diverse neuromuscular activation patterns across participants.

These results demonstrate the unique advantage of using a multivariate statistical pipeline for EEG–EMG integration. Compared to traditional approaches in archaeological sciences, this procedure more effectively handles the complexity of multimodal datasets and is better suited for understanding how neural and muscular systems interact. By identifying relevant EEG and EMG channels and using them to characterize variations across conditions/tasks and detect covariation patterns, this approach provides a replicable framework for studying other tool-related behaviors in experimental neuroarchaeology, and potentially in other fields interested in cognitive-motor dynamics. This could enhance the consistency and comparability of future experimental research on human brain-hand-tool interactions.

Future applications could extend the investigations into other fundamental aspects of tool use. For instance, this approach could be applied to explore the neuromechanical implications of other key stone tool industries, such as the Acheulean, which has often been associated with the gradual emergence of cumulative culture in hominins (de la Torre 2016; Shipton 2024). Moreover, focusing on Middle and Upper Palaeolithic industries associated with later hominin contexts (e.g., Neanderthals and modern humans) could offer new insights into the cognitive and motor demands of these populations’ behavioural practices. Beyond stone tools, this approach could also be extended to other forms of material culture, such as ornaments and engravings (Kuhn and Stiner 2007; Mellet et al. 2019), contributing to a broader understanding of the evolutionary factors underlying human manual skill and symbolic behavior.

A key limitation of this study is that it represents the first statistical integration of EEG and EMG in experimental neuroarchaeology. Therefore, this statistical approach requires validation across multiple datasets to further assess its broader robustness and generalizability in the field. Since EEG is highly susceptible to noise, rigorous data preprocessing is essential to ensure reliable results (as discussed in detail in Affinito et al. 2024; see also the dedicated step-by-step protocol by Eteson et al. 2024a, 2025) If not properly cleaned, the analysis may inadvertently capture artifacts rather than meaningful neural signals. To mitigate this issue, we strongly recommend implementing thorough preprocessing techniques, including artifact removal and careful selection of analysis parameters. Additionally, as described in our protocols, excluding individuals or channels exhibiting excessive noise and further increasing the sample size would improve statistical power and enhance the reliability of the findings.

Despite these challenges, as demonstrated in this paper’s case study, the integration of EEG and EMG represents a significant methodological advance in neuroarchaeology, enabling a more holistic and comprehensive exploration of tool-related brain-hand interactions. In the future, the application, development, and expansion of this statistical approach could offer new valuable insights into the complex relationships between brain function, manual dexterity, and the role of technology in the evolution of our lineage.

## Supplementary Information

Below is the link to the electronic supplementary material.

Supplementary file1 (PDF 775 KB)

## Data Availability

The R script and an example dataset used in the current study can be found in the GitHub repository (https://github.com/simonaaff/EEG-EMG-neuroarchaeology). The full dataset analyzed and generated during the experiment is available from the corresponding author upon reasonable request.

## References

[CR1] Affinito S, Eteson B, Cáceres LT et al (2024) Exploring the cognitive underpinnings of early hominin stone tool use through an experimental EEG approach. Sci Rep 14:26936. 10.1038/s41598-024-77452-0 39562652 10.1038/s41598-024-77452-0 PMC11576949

[CR2] Bell AJ, Sejnowski TJ (1995) An information-maximization approach to blind separation and blind deconvolution. Neural Comput 7:1129–1159. 10.1162/neco.1995.7.6.1129 7584893 10.1162/neco.1995.7.6.1129

[CR3] Brambilla C, Pirovano I, Mira RM et al (2021) Combined use of EMG and EEG techniques for neuromotor assessment in rehabilitative applications: a systematic review. Sensors 21:7014. 10.3390/s21217014 34770320 10.3390/s21217014 PMC8588321

[CR4] Braun DR, Aldeias V, Archer W et al (2019) Earliest known Oldowan artifacts at >2.58 Ma from Ledi-Geraru, Ethiopia, highlight early technological diversity. Proc Natl Acad Sci 116:11712–11717. 10.1073/pnas.1820177116 31160451 10.1073/pnas.1820177116 PMC6575601

[CR5] Bruner E (2019) Human paleoneurology: shaping cortical evolution in fossil hominids. J Comp Neurol 527:1753–1765. 10.1002/cne.24591 30520032 10.1002/cne.24591

[CR6] Bruner E (2017) The fossil evidence of human brain evolution. In: Kaas JH (ed) Evolutionary neuroscience, 2nd edn. Elsevier, pp 769–802

[CR7] Bruner E (2024) Cognitive archaeology, and the psychological assessment of extinct minds. J Comp Neurol 532:e25583. 10.1002/cne.25583 38289186 10.1002/cne.25583

[CR8] Bryche C, Lesourd M, Osiurak F (2024) From stone tools to fMRI, studying human cognitive evolution when the mind doesn’t fossilize. J Cult Cogn Sci 8:199–221. 10.1007/s41809-024-00154-6

[CR9] Cho J-H, Jeong J-H, Lee S-W (2022) NeuroGrasp: real-time eeg classification of high-level motor imagery tasks using a dual-stage deep learning framework. IEEE Trans Cybern 52:13279–13292. 10.1109/TCYB.2021.3122969 34748509 10.1109/TCYB.2021.3122969

[CR10] Crone N (1998) Functional mapping of human sensorimotor cortex with electrocorticographic spectral analysis. I. alpha and beta event- related desynchronization. Brain 121:2271–2299. 10.1093/brain/121.12.2271 9874480 10.1093/brain/121.12.2271

[CR11] de la Torre I (2016) The origins of the Acheulean: past and present perspectives on a major transition in human evolution. Philos Trans R Soc B Biol Sci 371:20150245. 10.1098/rstb.2015.0245 10.1098/rstb.2015.0245 PMC4920301 27298475

[CR12] Elsayed GF, Lara AH, Kaufman MT et al (2016) Reorganization between preparatory and movement population responses in motor cortex. Nat Commun 7:13239. 10.1038/ncomms13239 27807345 10.1038/ncomms13239 PMC5095296

[CR13] Eteson B, Affinito S, Karakostis FA (2024a) The mind & muscles: a protocol for the simultaneous measuring of cognitive and muscular activation during stone tool tasks using surface Electromyography and Electroencephalography. protocols.io. 10.17504/protocols.io.36wgqnxbygk5/v1

[CR14] Eteson B, Affinito S, Moos ET, Karakostis FA (2024b) “How handy was early hominin ‘know-how’?” an experimental approach exploring efficient early stone tool use. Am J Biol Anthropol. 10.1002/ajpa.25019 10.1002/ajpa.25019 39222398

[CR15] Eteson B, Affinito S, Karakostis FA (2025) The mind & muscles: Introducing a validated EEG/EMG protocol for recording cognitive-muscular interactions in experimental archaeology. PLoS ONE 20:e0324103. 10.1371/journal.pone.0324103 40408369 10.1371/journal.pone.0324103 PMC12101640

[CR16] Gola M, Magnuski M, Szumska I, Wróbel A (2013) EEG beta band activity is related to attention and attentional deficits in the visual performance of elderly subjects. Int J Psychophysiol 89:334–341. 10.1016/j.ijpsycho.2013.05.007 23688673 10.1016/j.ijpsycho.2013.05.007

[CR17] Good P (2000) permutation tests: a practical guide to resampling methods for testing hypotheses. Springer New York, New York

[CR18] Guerrero-Mendez CD, Ruiz-Olaya AF (2022) Coherence-based connectivity analysis of EEG and EMG signals during reach-to-grasp movement involving two weights. Brain Comput Interfaces 9:140–154. 10.1080/2326263X.2022.2029308

[CR19] Hamrick MW, Churchill SE, Schmitt D, Hylander WL (1998) EMG of the human flexor pollicis longus muscle: implications for the evolution of hominid tool use. J Hum Evol 34:123–136. 10.1006/jhev.1997.0177 9503091 10.1006/jhev.1997.0177

[CR20] Hecht EE, Gutman DA, Khreisheh N et al (2015) Acquisition of Paleolithic toolmaking abilities involves structural remodeling to inferior frontoparietal regions. Brain Struct Funct 220:2315–2331. 10.1007/s00429-014-0789-6 24859884 10.1007/s00429-014-0789-6

[CR21] Hecht EE, Pargeter J, Khreisheh N, Stout D (2023) Neuroplasticity enables bio-cultural feedback in Paleolithic stone-tool making. Sci Rep 13:2877. 10.1038/s41598-023-29994-y 36807588 10.1038/s41598-023-29994-y PMC9938911

[CR22] Helwig NE (2018) eegkit: Toolkit for electroencephalography data. http://CRAN.R-project.org/package=eegkit. Accessed 26 Jan 2025

[CR23] Jolliffe IT (2002) Principal Component Analysis, 2nd edn. Springer-Verlag, Heidelberg

[CR24] Kaufman MT, Churchland MM, Ryu SI, Shenoy KV (2014) Cortical activity in the null space: permitting preparation without movement. Nat Neurosci 17:440–448. 10.1038/nn.3643 24487233 10.1038/nn.3643 PMC3955357

[CR25] Key AJM, Farr I, Hunter R, Winter SL (2020) Muscle recruitment and stone tool use ergonomics across three million years of Palaeolithic technological transitions. J Hum Evol 144:102796. 10.1016/j.jhevol.2020.102796 32470872 10.1016/j.jhevol.2020.102796

[CR26] Key A, Farr I, Hunter R et al (2021) Why invent the handle? electromyography (EMG) and efficiency of use data investigating the prehistoric origin and selection of hafted stone knives. Archaeol Anthropol Sci 13:162. 10.1007/s12520-021-01421-1

[CR27] Kilavik BE, Zaepffel M, Brovelli A et al (2013) The ups and downs of beta oscillations in sensorimotor cortex. Exp Neurol 245:15–26. 10.1016/j.expneurol.2012.09.014 23022918 10.1016/j.expneurol.2012.09.014

[CR28] Kivell TL, Baraki N, Lockwood V et al (2023) Form, function and evolution of the human hand. Am J Biol Anthropol 181:6–57. 10.1002/ajpa.24667

[CR29] Kuhn SL, Stiner MC (2007) Paleolithic ornaments: implications for cognition, demography and identity. Diogenes 54:40–48. 10.1177/0392192107076870

[CR30] Lee T-W, Girolami M, Sejnowski TJ (1999) Independent component analysis using an extended infomax algorithm for mixed Subgaussian and Supergaussian sources. Neural Comput 11:417–441. 10.1162/089976699300016719 9950738 10.1162/089976699300016719

[CR31] Li H, Huang G, Lin Q et al (2018) Combining movement-related cortical potentials and event-related desynchronization to study movement preparation and execution. Front Neurol. 10.3389/fneur.2018.00822 10.3389/fneur.2018.00822 PMC6182054 30344504

[CR32] Logothetis NK (2008) What we can do and what we cannot do with fMRI. Nature 453:869–878. 10.1038/nature06976 18548064 10.1038/nature06976

[CR33] Macchi R, Daver G, Brenet M et al (2021) Biomechanical demands of percussive techniques in the context of early stone toolmaking. J R Soc Interface 18:20201044. 10.1098/rsif.2020.1044 34034530 10.1098/rsif.2020.1044 PMC8150015

[CR34] Marino M, Mantini D (2024) Human brain imaging with high-density electroencephalography: techniques and applications. J Physiol. 10.1113/JP286639 10.1113/JP286639 PMC12810243 39173191

[CR35] Marzke MW, Shackley MS (1986) Hominid hand use in the Pliocene and Pleistocene: evidence from experimental archaeology and comparative morphology. J Hum Evol 15:439–460. 10.1016/S0047-2484(86)80027-6

[CR36] Marzke MW, Toth N, Schick K et al (1998) EMG study of hand muscle recruitment during hard hammer percussion manufacture of Oldowan tools. Am J Phys Anthropol 105:315–332. 10.1002/(SICI)1096-8644(199803)105:3%3c315::AID-AJPA3%3e3.0.CO;2-Q 9545075 10.1002/(SICI)1096-8644(199803)105:3<315::AID-AJPA3>3.0.CO;2-Q

[CR37] Mellet E, Salagnon M, Majkić A et al (2019) Neuroimaging supports the representational nature of the earliest human engravings. R Soc Open Sci 6:190086. 10.1098/rsos.190086 31417715 10.1098/rsos.190086 PMC6689598

[CR38] Profico A, Zeppilli C, Micarelli I et al (2021) Morphometric maps of bilateral asymmetry in the human humerus: an implementation in the r package morphomap. Symmetry (Basel) 13:1711. 10.3390/sym13091711

[CR39] Putt SS, Wijeakumar S, Franciscus RG, Spencer JP (2017) The functional brain networks that underlie early stone age tool manufacture. Nat Hum Behav. 10.1038/s41562-017-0102

[CR40] Putt SSJ, Wijeakumar S, Spencer JP (2019) Prefrontal cortex activation supports the emergence of early stone age toolmaking skill. Neuroimage 199:57–69. 10.1016/j.neuroimage.2019.05.056 31128246 10.1016/j.neuroimage.2019.05.056

[CR41] R Core Team (2021) R: a language and environment for statistical computing. https://www.R-project.org/. Accessed 26 Jan 2025

[CR42] Rohlf FJ, Corti M (2000) Use of two-block partial least-squares to study covariation in shape. Syst Biol 49:740–753. 10.1080/106351500750049806 12116437 10.1080/106351500750049806

[CR43] Rolian C, Lieberman DE, Zermeno JP (2011) Hand biomechanics during simulated stone tool use. J Hum Evol 61:26–41. 10.1016/j.jhevol.2011.01.008 21420144 10.1016/j.jhevol.2011.01.008

[CR44] Salagnon M, D’Errico F, Mellet E (2020) Neuroimaging and neuroarchaeology: a window on cognitive evolution. Intellect Rev De L’assoc Pour La Recherche Cognit 73:67–91. 10.3406/intel.2020.1965

[CR45] Schlager S (2017) Morpho and Rvcg—shape analysis in R. In: Zheng G, Li S, Szekely G (eds) Statistical shape and deformation analysis. Academic Press, Cambridge, pp 217–256

[CR46] Schneider S, Rouffet DM, Billaut F, Strüder HK (2013) Cortical current density oscillations in the motor cortex are correlated with muscular activity during pedaling exercise. Neuroscience 228:309–314. 10.1016/j.neuroscience.2012.10.037 23103214 10.1016/j.neuroscience.2012.10.037

[CR47] Semaw S, Rogers MJ, Quade J et al (2003) 2.6-Million-year-old stone tools and associated bones from OGS-6 and OGS-7, Gona, Afar. Ethiopia J Hum Evol 45:169–177. 10.1016/S0047-2484(03)00093-9 14529651 10.1016/s0047-2484(03)00093-9

[CR48] Shipton C (2024) Was culture cumulative in the Palaeolithic? Phenomenol Cogn Sci. 10.1007/s11097-024-10005-y

[CR49] Stegeman D, Hermens H (2007) Standards for surface electromyography, the European project surface EMG for non-invasive assessment of muscles (SENIAM). Enschede Roessingh Res Dev 10:8–12

[CR50] Stout D, Chaminade T (2007) The evolutionary neuroscience of tool making. Neuropsychologia 45:1091–1100. 10.1016/j.neuropsychologia.2006.09.014 17070875 10.1016/j.neuropsychologia.2006.09.014

[CR51] Stout D, Hecht E (2015) Neuroarchaeology. In: Bruner E (ed) Human paleoneurology. Springer, Cham, pp 145–175

[CR52] Stout D, Toth N, Schick K et al (2000) Stone tool-making and brain activation: position emission tomography (PET) studies. J Archaeol Sci 27:1215–1223. 10.1006/jasc.2000.0595

[CR53] Stout D, Toth N, Schick K, Chaminade T (2008) Neural correlates of early stone age toolmaking: technology, language and cognition in human evolution. Philos Trans R Society B Biol Sci 363:1939–1949. 10.1098/rstb.2008.0001 10.1098/rstb.2008.0001 PMC2606694 18292067

[CR54] Stout D, Passingham R, Frith C et al (2011) Technology, expertise and social cognition in human evolution. Eur J Neurosci 33:1328–1338. 10.1111/j.1460-9568.2011.07619.x 21375598 10.1111/j.1460-9568.2011.07619.x

[CR55] Stout D, Hecht E, Khreisheh N et al (2015) Cognitive demands of lower Paleolithic toolmaking. PLoS ONE 10:e0121804. 10.1371/journal.pone.0121804 25875283 10.1371/journal.pone.0121804 PMC4398452

[CR56] Stout D (2023) Experimental neuroarchaeology of visuospatial behavior. Cognitive archaeology, body cognition, and the evolution of visuospatial perception. Elsevier, Amsterdam, pp 195–211

[CR57] Tharwat A, Gaber T, Ibrahim A, Hassanien AE (2017) Linear discriminant analysis: a detailed tutorial. AI Commun 30:169–190. 10.3233/AIC-170729

[CR58] Turella L, Tucciarelli R, Oosterhof NN et al (2016) Beta band modulations underlie action representations for movement planning. Neuroimage 136:197–207. 10.1016/j.neuroimage.2016.05.027 27173760 10.1016/j.neuroimage.2016.05.027

[CR59] Uomini NT, Meyer GF (2013) Shared brain lateralization patterns in language and acheulean stone tool production: a functional transcranial doppler ultrasound study. PLoS ONE 8:e72693. 10.1371/journal.pone.0072693 24023634 10.1371/journal.pone.0072693 PMC3758346

[CR60] Venables WN, Ripley BD (2002) Modern applied statistics with S, 4th edn. Springer, New York

[CR61] Warriner CL, Fageiry S, Saxena S et al (2022) Motor cortical influence relies on task-specific activity covariation. Cell Rep 40:111427. 10.1016/j.celrep.2022.111427 36170841 10.1016/j.celrep.2022.111427 PMC9536049

[CR62] Williams EM, Gordon AD, Richmond BG (2010) Upper limb kinematics and the role of the wrist during stone tool production. Am J Phys Anthropol 143:134–145. 10.1002/ajpa.21302 20734439 10.1002/ajpa.21302

[CR63] Woolson RF (2005) Wilcoxon signed-rank test. In: Encyclopedia of Biostatistics. Wiley, 10.1002/0470011815.b2a15177

[CR64] Wynn T, Coolidge FL (2016) Archeological insights into hominin cognitive evolution. Evol Anthropol Issues News Rev 25:200–213. 10.1002/evan.21496 10.1002/evan.21496 27519459

[CR65] Yang J, Rahardja S, Fränti P (2019) Outlier detection: how to threshold outlier scores? In: proceedings of the international conference on artificial intelligence, information processing and cloud computing. ACM, New York. pp 1–6

[CR66] Yao D (2001) A method to standardize a reference of scalp EEG recordings to a point at infinity. Physiol Meas 22:693–711. 10.1088/0967-3334/22/4/305 11761077 10.1088/0967-3334/22/4/305

[CR67] Yu C, Zhan J, Xu L et al (2025) Motor control performance-related modulation of beta-band EEG–sEMG coherence differs between general and local muscular exercise-induced fatigue. Eur J Appl Physiol. 10.1007/s00421-025-05714-4 10.1007/s00421-025-05714-4 39909897

[CR68] Zaepffel M, Trachel R, Kilavik BE, Brochier T (2013) Modulations of EEG beta power during planning and execution of grasping movements. PLoS ONE 8:e60060. 10.1371/journal.pone.0060060 23555884 10.1371/journal.pone.0060060 PMC3605373

