# Supplementary material for: Integrating EEG and EMG data: a novel statistical pipeline for investigating brain-muscle interaction in experimental neuroarchaeology
Source: Brain Struct Funct. 2025 Jun 16;230(6):101. doi: 10.1007/s00429-025-02961-1 (PMC12170762; doi:10.1007/s00429-025-02961-1)
Supplement: Supplementary file 1 — Supplementary file1 (PDF 775 KB) [file 429_2025_2961_MOESM1_ESM.pdf]

SUPPLEMENTARY INFORMATION

Brain Structure and Function

Integrating EEG and EMG data: a novel statistical pipeline for investigating brain-muscle interaction in experimental neuroarchaeology

Simona Affinito[1], Brienna Eteson[1], Fotios Alexandros Karakostis[1,2,3] *

[1] DFG Center for Advanced Studies "Words, Bones, Genes, Tools", Department of Geosciences, Eberhard Karls University of Tübingen, Tübingen, Germany

[2] Paleoanthropology, Senckenberg Centre for Human Evolution and Palaeoenvironment, Department of Geosciences, Eberhard Karls University of Tübingen, Tübingen, Germany

[3] Integrative Prehistory and Archaeological Science, University of Basel, Basel, Switzerland

*Corresponding author fotios-alexandros.karakostis@uni-tuebingen.de

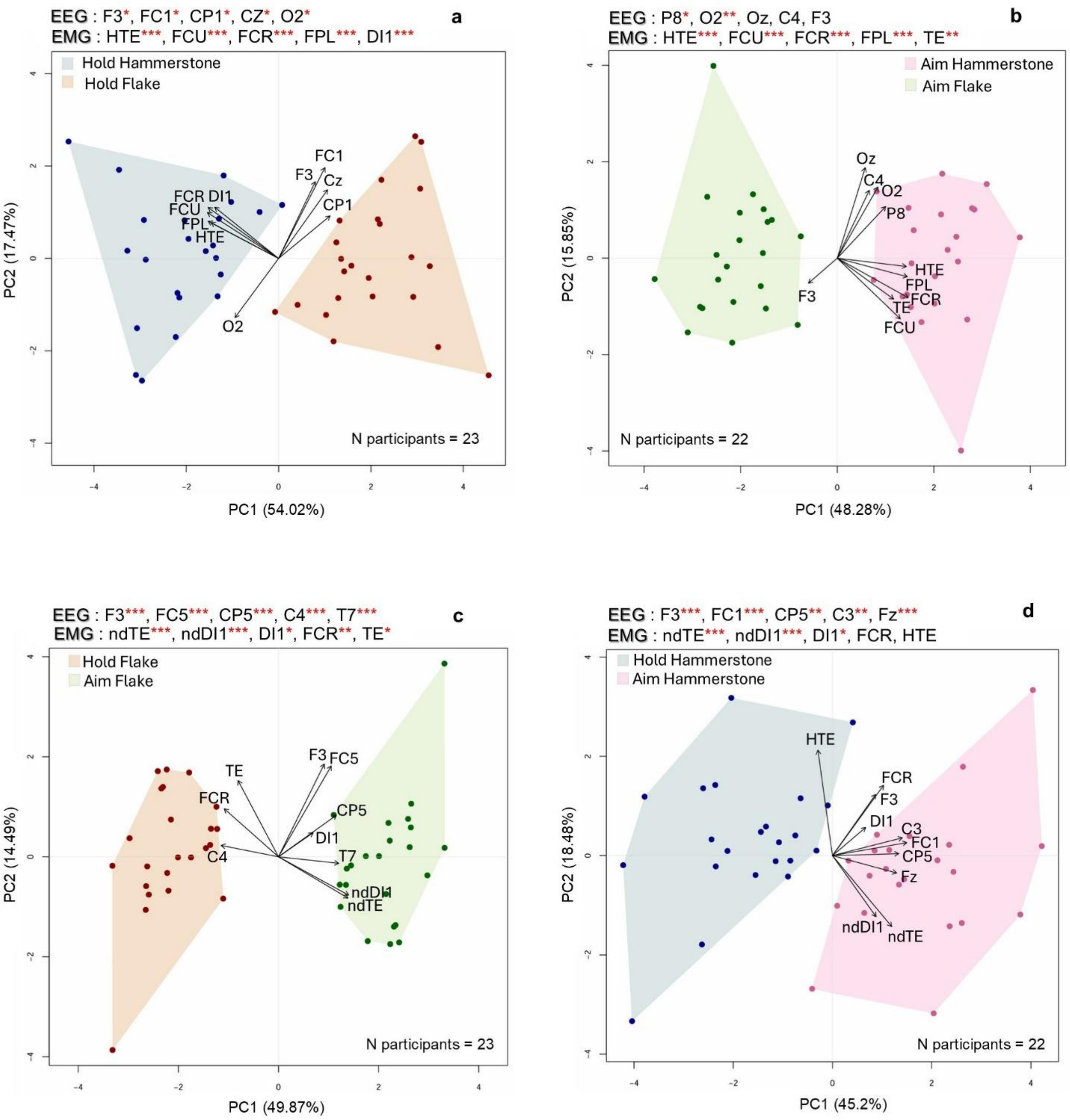

Fig. S1 PCA plots illustrating comparisons within the same experimental condition – Hold vs. Hold (a) and Aim vs. Aim (b) in both flake and hammerstone use – as well as between different conditions – Hold vs. Aim for flake use (c) and hammerstone use (d). The analysis was performed using the 10 EEG and EMG channels selected based on the highest absolute Z scores in the Wilcoxon signed-rank test selection approach. The plots reflect PCA results after outlier removal. Muscle abbreviations: FCR= *flexor carpi radialis*; FCU= *flexor carpi ulnaris*; FPL= *flexor pollicis longus*; TE= thenar eminence muscle group; HTE= hypothenar eminence muscle group; DI1= first dorsal *interosseus*; ndDI1= non-dominant first dorsal *interosseus*; ndTE= non-dominant thenar eminence group. All other channels correspond to EEG electrodes following the 10-20 international system (32-channel configuration).

Table S1 Wilcoxon signed-rank test results for hammerstone "Hold" vs flake "Hold" and hammerstone "Aim" vs flake "Aim" paired comparisons, excluding PCA outliers. Significant p-values are highlighted in bold. Muscle abbreviations: FCR= *flexor carpi radialis*; FCU= *flexor carpi ulnaris*; FPL= *flexor pollicis longus*; TE= thenar eminence muscle group; HTE= hypothenar eminence muscle group; DI1= first dorsal *interosseus*; ndDI1= non-dominant first dorsal *interosseus*; ndTE= non-dominant thenar eminence group. All other channels correspond to EEG electrodes following the 10-20 international system (32-channel configuration).

	**H-Hold vs H-Aim (22 participants)**		**H-Aim vs F-Aim (22 participants)**	
Channels	**Z scores**	**p-value**	**Z scores**	**p-value**
Fz	-3.3440	**0.0008*** **	-0.1299	0.8967
F3	-3.9933	**6.52E-05*** **	-1.6557	0.0978
F7	-2.2077	**0.0273* **	-0.3247	0.7454
FC5	-2.8245	**0.0047** **	-1.1038	0.2697
FC1	-3.3764	**0.0007*** **	-0.1299	0.8967
C3	-3.1492	**0.0016** **	-0.7467	0.4552
T7	-2.1427	**0.0321* **	-0.1299	0.8967
CP5	-3.1492	**0.0016** **	-0.5844	0.5590
CP1	-2.7596	**0.0058** **	-0.2922	0.7701
Pz	-1.2662	0.2055	-0.6493	0.5161
P3	-2.3375	**0.0194* **	-0.7792	0.4359
P7	-2.5648	**0.0103* **	-0.7792	0.4359
O1	-0.7467	0.4552	-1.4934	0.1353
Oz	-0.3571	0.7210	-1.9155	0.0554
O2	0.0000	1.0000	-2.5973	**0.0094** **
P4	-1.0064	0.3142	-1.6557	0.0978
P8	-0.9740	0.3301	-2.4674	**0.0136* **
CP6	-2.0778	**0.0377* **	-1.4934	0.1353
CP2	-0.0649	0.9482	-0.4545	0.6495
Cz	-2.0453	**0.0408* **	-0.1623	0.8710
C4	-1.9155	0.0554	-1.8181	0.0691
T8	-0.6493	0.5161	-0.2597	0.7951
FC6	-0.4221	0.6730	-0.5844	0.5590
FC2	-1.3311	0.1832	-0.2597	0.7951
F4	-0.0649	0.9482	-0.6168	0.5373
F8	-0.6168	0.5373	-0.4870	0.6263
ndTE	-4.0907	**4.30E-05*** **	-1.9804	**0.0477* **
FCU	-0.5844	0.5590	-3.9933	**0.0001*** **
FCR	-1.8830	0.0597	-4.0907	**4.30E-05*** **
FPL	-0.1623	0.8710	-4.0582	**4.95E-05*** **
TE	-0.1948	0.8456	-3.2466	**0.0012** **
DI1	-2.0453	**0.0408* **	-3.1492	**0.0016** **
HTE	-1.1038	0.2697	-4.0582	**4.95E-05*** **
ndDI1	-4.0907	**4.30E-05*** **	-1.2662	0.2055

p<0.001*** p<0.01** p<0.05*

Table S2 LDA-based permutation test results for hammerstone "Hold" vs flake "Hold", hammerstone "Hold" vs hammerstone "Aim", and hammerstone "Aim" vs flake "Aim" paired comparisons, excluding PCA outliers. Muscle abbreviations: FCR= *flexor carpi radialis*; FCU= *flexor carpi ulnaris*; FPL= *flexor pollicis longus*; TE= thenar eminence muscle group; HTE= hypothenar eminence muscle group; DI1= first dorsal *interosseus*; ndDI1= non-dominant first dorsal *interosseus*; ndTE= non-dominant thenar eminence group. All other channels correspond to EEG electrodes following the 10-20 international system (32-channel configuration).

n=201376 permutations		n=278256 permutations		n=278256 permutations	
**H-Hold vs F-Hold (22 participants)**		**H-Hold vs H-Aim (19 participants)**		**H-Aim vs F-Aim (20 participants)**	
**Channels**	**Accuracy (%)**	**Channels**	**Accuracy (%)**	**Channels**	**Accuracy (%)**
FCR	81.6362	ndTE	94.2314	FCR	89.1047
HTE	79.8313	ndDI1	75.5965	HTE	83.5081
FCU	78.9196	C4	68.6416	FCU	81.7075
FPL	78.2412	FC1	68.6257	FPL	81.4610
O2	75.0917	P7	68.4930	TE	77.2509
Oz	73.7999	F3	68.0919	ndTE	75.9894
DI1	72.6153	FC5	67.9209	O2	75.9603
CP5	72.6091	Fz	67.6200	P8	74.6948
CP6	72.3880	FCR	67.3023	Oz	74.5871
F3	72.3353	CP1	67.1913	DI1	73.7045
P4	72.3221	C3	67.1130	P4	73.6419
TE	72.0726	FC2	67.0633	CP6	73.1397
FC1	72.0129	CP5	66.8457	C4	72.7374
Cz	71.8836	CP2	66.7114	Pz	72.7355
P8	71.6795	DI1	66.5653	ndDI1	72.6126
O1	71.5076	P4	66.5363	T8	72.5820
CP1	71.3456	FC6	66.4829	F3	72.3310
T8	71.2959	P8	66.3514	CP1	72.2980
P3	71.1991	T7	66.2637	CP5	72.2675
C3	71.0732	P3	66.1935	CP2	72.1897
Fz	71.0431	T8	66.1778	O1	72.0827
FC2	71.0104	CP6	66.0416	C3	72.0819
P7	70.9160	F7	66.0279	F4	72.0467
FC6	70.9113	Cz	66.0253	FC6	72.0320
F4	70.8673	FPL	65.8688	FC2	72.0166
CP2	70.8573	F4	65.8665	FC1	71.9691
F8	70.8387	HTE	65.8647	Cz	71.9658
T7	70.8192	Pz	65.8589	Fz	71.9293
FC5	70.7266	F8	65.7967	P3	71.8936
Pz	70.7176	O1	65.7234	FC5	71.8345
C4	70.5917	TE	65.6940	F8	71.7150
F7	70.4562	O2	65.6698	F7	71.7147
		FCU	65.5756	T7	71.6817
		Oz	65.4477	P7	71.6750

Table S3 Principal Component Analysis statistics and loadings values for the paired comparison of the two "Hold" conditions (22 participants). Selection of channels based on LDA-based permutation test. Significant p-values are highlighted in bold. Muscle abbreviations: FCR= *flexor carpi radialis*; FCU= *flexor carpi ulnaris*; FPL= *flexor pollicis longus*; HTE= hypothenar eminence muscle group; DI1= first dorsal *interosseus*. All other channels correspond to EEG electrodes following the 10-20 international system (32-channel configuration).

Hold vs Hold

				PCA Loadings									
	**Eigenvalues**	**% Variance**	**Wilcoxon test PC scores (p-value)**	**Oz**	**O2**	**CP6**	**F3**	**CP5**	**HTE**	**FCR**	**FCU**	**FPL**	**DI1**
PC1	224.0178	50.1840	**4.77E-07*****	0.2612	0.2920	0.1408	-0.2255	0.1130	0.3842	0.4147	0.3908	0.4104	0.3497
PC2	130.0850	16.9221	0.7024	0.2090	0.3491	0.3978	-0.5333	-0.5156	-0.1092	-0.1823	-0.2523	-0.0353	-0.1257
PC3	99.4643	9.8931	0.7990	0.4974	-0.3011	-0.4618	0.0546	-0.5019	0.0496	-0.0614	0.0114	-0.1243	0.4147
PC4	84.8148	7.1935	0.7990	0.1225	0.3336	-0.7228	-0.3716	0.2514	-0.0723	0.0837	-0.1946	0.0867	-0.3041
PC5	76.6228	5.8711	1.0000	0.7188	-0.1657	0.2743	-0.0120	0.5388	-0.1461	-0.0823	-0.0826	-0.2202	-0.0820
PC6	61.8591	3.8266	0.6789	0.0632	0.6033	-0.0343	0.3891	0.0154	-0.5861	-0.0739	0.1216	-0.0292	0.3387
PC7	52.8313	2.7911	0.8486	0.2762	0.2463	0.0359	0.6073	-0.2150	0.3476	0.1728	-0.3148	0.1763	-0.4072
PC8	42.4626	1.8031	0.7990	0.0250	-0.3500	0.0682	-0.0207	-0.0371	-0.4817	0.2673	-0.3430	0.6679	0.0565
PC9	33.9646	1.1536	0.7502	0.0945	-0.1144	0.0032	-0.0158	-0.2567	-0.3384	0.5085	0.5243	-0.2175	-0.4654
PC10	19.0207	0.3618	0.9746	0.1379	-0.0565	-0.0627	0.0512	-0.0473	-0.0238	-0.6419	0.4821	0.4833	-0.3009

p<0.001*** p<0.01** p<0.05*

Table S4 Principal Component Analysis statistics and loadings values for the paired comparison Hammerstone "Aim" vs Flake "Aim" (20 participants). Selection of channels based on LDA-based permutation test. Significant p-values are highlighted in bold. Muscle abbreviations: FCR= *flexor carpi radialis*; FCU= *flexor carpi ulnaris*; FPL= *flexor pollicis longus*; TE= thenar eminence muscle group; HTE= hypothenar eminence muscle group. All other channels correspond to EEG electrodes following the 10-20 international system (32-channel configuration).

Aim vs Aim

				PCA Loadings									
	**Eigenvalues**	**% Variance**	**Wilcoxon test PC scores (p-value)**	**Oz**	**O2**	**P4**	**CP6**	**P8**	**HTE**	**FCR**	**FCU**	**FPL**	**TE**
PC1	212.9173	45.3338	**1.91E-06*** **	0.1714	0.2128	0.2094	0.1492	0.2611	0.4129	0.4338	0.3857	0.4181	0.3299
PC2	151.2510	22.8769	0.7012	0.3293	0.2951	0.4854	0.5193	0.2631	-0.0538	-0.1975	-0.2923	-0.1137	-0.2997
PC3	99.7364	9.9474	0.6477	0.4994	0.6201	-0.1593	-0.3899	-0.3101	-0.0802	-0.0351	-0.2192	0.0691	0.1786
PC4	91.4914	8.3707	0.9854	0.6010	-0.4040	-0.3369	-0.0948	0.5040	0.0845	-0.1067	-0.0263	-0.2304	0.1635
PC5	70.9558	5.0347	0.8983	0.2884	-0.2938	0.0697	0.2417	-0.6554	0.5561	-0.0582	0.0080	-0.1561	-0.0262
PC6	63.5534	4.0390	0.5459	0.1426	-0.0857	-0.2644	-0.1173	0.0410	0.1341	0.2707	-0.1423	0.4493	-0.7573
PC7	47.2021	2.2280	0.4980	0.0886	-0.3171	0.6995	-0.6088	0.0277	0.0244	-0.0068	-0.1440	0.0976	-0.0090
PC8	37.0143	1.3701	0.9563	0.3195	-0.0255	0.1308	-0.0136	-0.1785	-0.4658	0.1858	0.6889	-0.2236	-0.2684
PC9	25.3946	0.6449	0.7012	0.1938	-0.3343	-0.0020	0.2980	-0.2062	-0.4747	-0.1343	-0.1253	0.6195	0.2767
PC10	12.4324	0.1546	0.8983	0.0224	-0.1153	0.0243	0.1238	-0.0781	-0.2071	0.7943	-0.4310	-0.2912	0.1408

p<0.001*** p<0.01** p<0.05*

Table S5 Principal Component Analysis statistics and loadings values for the paired comparison Flake “Hold” vs Flake “Aim” (23 participants). Selection of channels based on LDA-based permutation test. Significant p-values are highlighted in bold. Muscle abbreviations: FCU= *flexor carpi ulnaris*; TE= thenar eminence muscle group; DI1= first dorsal *interosseus*; ndDI1= non-dominant first dorsal *interosseus*; ndTE= non-dominant thenar eminence group. All other channels correspond to EEG electrodes following the 10-20 international system (32-channel configuration).

Flake Hold vs Flake Aim

				PCA Loadings									
	**Eigenvalues**	**% Variance**	**Wilcoxon test PC scores (p-value)**	**F3**	**CP1**	**C4**	**FC1**	**C3**	**ndTE**	**ndDI1**	**FCU**	**TE**	**DI1**
PC1	208.5350	43.4868	**2.38E-07*** **	0.2728	0.2372	-0.3762	0.2916	0.4033	0.4273	0.3735	0.2437	-0.2198	0.2243
PC2	138.1117	19.0749	0.3931	0.1946	0.5147	0.1717	0.4011	0.3542	-0.2173	-0.3497	-0.4081	0.2128	-0.0034
PC3	111.0960	12.3423	0.9168	0.0784	-0.2844	-0.0263	0.2283	-0.0273	-0.1138	-0.0256	0.3088	0.6944	0.5179
PC4	93.3048	8.7058	0.9406	0.5703	-0.2810	-0.1434	0.2951	-0.1065	-0.1489	-0.0501	0.2158	0.0643	-0.6336
PC5	79.3531	6.2969	0.3765	0.6403	-0.0752	-0.0782	-0.4102	-0.1513	-0.0540	0.2182	-0.5021	0.0297	0.2892
PC6	66.1675	4.3781	0.9881	0.3346	0.1183	0.7986	-0.0542	-0.0427	0.0981	-0.0066	0.3975	-0.2094	0.1416
PC7	49.9404	2.4940	0.9881	0.0608	-0.4207	-0.1026	0.3094	-0.0259	-0.1010	-0.4774	-0.0972	-0.5632	0.3842
PC8	42.7264	1.8255	1.0000	0.1659	0.3031	-0.3498	-0.5165	0.0983	-0.1085	-0.5419	0.4172	0.0066	0.0482
PC9	31.8325	1.0133	0.4452	0.0512	-0.1780	0.0769	-0.0553	-0.0046	0.8268	-0.4043	-0.1939	0.2395	-0.1213
PC10	19.5513	0.3823	0.8462	0.0238	0.4458	-0.1586	0.2796	-0.8153	0.1305	-0.0478	0.0316	-0.0251	0.1081

p<0.001*** p<0.01** p<0.05*

Table S6 Principal Component Analysis statistics and loadings values for the paired comparison Hammerstone "Hold" vs Hammerstone "Aim" (19 participants). Selection of channels based on LDA-based permutation test. Significant p-values are highlighted in bold. Muscle abbreviations: FCR= *flexor carpi radialis*; FPL= *flexor pollicis longus*; DI1= first dorsal *interosseus*; ndDI1= non-dominant first dorsal *interosseus*; ndTE= non-dominant thenar eminence group. All other channels correspond to EEG electrodes following the 10-20 international system (32-channel configuration).

Hammerstone Hold vs Hammerstone Aim													
				PCA Loadings									
	Eigenvalues	% Variance	Wilcoxon test PC scores (p-value)	F3	FC1	C4	FC5	P7	ndTE	ndDI1	FCR	FPL	DI1
PC1	221.1200	48.8940	**3.81E-06*****	0.3529	0.3644	-0.0591	0.3384	0.3393	0.3655	0.4043	0.3271	0.1527	0.2866
PC2	139.7326	19.5252	0.5678	0.2996	0.0691	-0.2300	0.2078	0.3285	-0.0567	0.0800	-0.4625	-0.6093	-0.3267
PC3	104.5266	10.9258	0.4900	0.1734	-0.3699	-0.7457	0.1306	-0.0573	-0.2763	-0.0775	0.0100	0.1347	0.3950
PC4	93.9063	8.8184	0.0602	0.3330	-0.2884	0.5278	-0.0968	0.4151	-0.4457	-0.0792	0.0326	-0.0681	0.3675
PC5	68.7355	4.7246	0.6507	0.0522	-0.2004	-0.1305	-0.7385	-0.0095	0.2548	0.4720	0.0483	-0.2851	0.1528
PC6	53.4315	2.8549	0.7381	0.3021	0.3730	-0.2045	-0.4662	0.2519	-0.2742	-0.0956	-0.0495	0.4850	-0.3536
PC7	43.6700	1.9071	0.6794	0.3154	-0.4773	0.1455	0.2101	-0.2539	-0.0806	0.4851	0.0405	0.2750	-0.4732
PC8	30.8524	0.9519	0.9217	0.4985	0.2300	0.0170	-0.0569	-0.5145	-0.1287	-0.2411	0.4607	-0.3758	-0.0479
PC9	28.1385	0.7918	0.5949	0.3831	0.1102	0.1669	-0.0405	-0.3800	0.2685	-0.0748	-0.6621	0.2124	0.3257
PC10	24.6247	0.6064	0.9843	0.2366	-0.4127	-0.0268	-0.0642	0.2536	0.5941	-0.5356	0.1447	0.0656	-0.1986

$p<0.001$*** $p<0.01$** $p<0.05$*

Table S7 Principal Component Analysis statistics and loadings values for the paired comparison Hammerstone "Hold" vs flake "Hold" (23 participants). Selection of channels based on Wilcoxon signed-rank test. Significant p-values are highlighted in bold. Muscle abbreviations: FCR= *flexor carpi radialis*; FCU= *flexor carpi ulnaris*; FPL= *flexor pollicis longus*; HTE= hypothenar eminence muscle group; DI1= first dorsal *interosseus*. All other channels correspond to EEG electrodes following the 10-20 international system (32-channel configuration).

Hold vs Hold

				PCA Loadings									
	**Eigenvalues**	**% Variance**	**Wilcoxon test PC scores (p-value)**	**FCU**	**FCR**	**FPL**	**HTE**	**DI1**	**O2**	**CP1**	**FC1**	**Cz**	**F3**
PC1	232.4233	54.0206	**4.77E-07*****	-0.3856	-0.3818	-0.3814	-0.3661	-0.3457	-0.2362	0.2786	0.2511	0.2655	0.1990
PC2	132.1691	17.4687	0.5202	0.2482	0.2758	0.2008	0.1940	0.2771	-0.3200	0.2300	0.4914	0.3719	0.4153
PC3	111.5360	12.4403	0.4100	-0.1353	0.1195	0.2199	0.0771	-0.1082	0.4601	0.5267	0.1910	0.3230	-0.5210
PC4	70.3472	4.9487	0.8229	0.0438	-0.0428	-0.1416	0.1014	0.2737	-0.6461	0.3244	-0.3934	0.0912	-0.4531
PC5	62.3007	3.8814	0.9643	-0.1431	-0.0152	0.1522	0.2321	-0.1985	-0.2473	0.2525	0.4114	-0.7472	-0.1020
PC6	53.8672	2.9017	0.4820	0.0878	-0.1436	0.0480	-0.6476	0.6291	0.1509	0.1765	0.1723	-0.2612	-0.0490
PC7	40.8386	1.6678	0.4452	0.1863	0.4907	0.3031	-0.5295	-0.4415	-0.1755	0.1930	-0.2718	-0.0713	0.1017
PC8	38.6355	1.4927	0.4634	0.0490	0.0262	-0.2886	0.2344	0.0862	0.3037	0.5823	-0.3886	-0.2017	0.4777
PC9	29.8814	0.8929	0.7768	0.6891	0.0818	-0.5837	-0.0393	-0.2067	0.0751	0.0058	0.2636	-0.0555	-0.2352
PC10	16.8905	0.2853	0.9168	0.4788	-0.7022	0.4522	0.0438	-0.1920	-0.0077	0.1244	-0.1049	0.0569	0.0676

$p<0.001$*** $p<0.01$** $p<0.05$*

Table S8 Principal Component Analysis statistics and loadings values for the paired comparison Hammerstone "Aim" vs Flake "Aim" (22 participants). Selection of channels based on Wilcoxon signed-rank test. Significant p-values are highlighted in bold. Muscle abbreviations: FCR= *flexor carpi radialis*; FCU= *flexor carpi ulnaris*; FPL= *flexor pollicis longus*; TE= thenar eminence muscle group; HTE= hypothenar eminence muscle group. All other channels correspond to EEG electrodes following the 10-20 international system (32-channel configuration).

Aim vs Aim

				PCA Loadings									
	**Eigenvalues**	**% Variance**	**Wilcoxon test PC scores (p-value)**	**Oz**	**O2**	**C4**	**F3**	**P8**	**HTE**	**FCR**	**FCU**	**FPL**	**TE**
PC1	219.7173	48.2757	**4.77E-07*** **	0.1656	0.2408	0.1898	-0.1700	0.2865	0.4090	0.4206	0.3732	0.4157	0.3337
PC2	125.8811	15.8461	0.7502	0.5362	0.4216	0.4023	-0.1479	0.3061	-0.0491	-0.2347	-0.3591	-0.1103	-0.2423
PC3	114.5221	13.1153	1.0000	0.1209	0.3143	-0.4935	-0.7014	-0.3767	-0.0534	0.0205	-0.0406	0.0588	-0.0284
PC4	83.3659	6.9499	0.8486	0.6983	-0.4969	-0.2861	0.0519	0.0067	0.1747	-0.0585	-0.0226	-0.1983	0.3273
PC5	73.8041	5.4470	0.8987	0.1773	0.1366	-0.5514	0.3055	0.3877	-0.1299	0.2349	0.2055	0.0901	-0.5290
PC6	61.6467	3.8003	0.8987	0.2637	0.4213	0.0286	0.5615	-0.6376	0.0535	0.1164	-0.0149	0.0915	0.0727
PC7	58.8025	3.4577	0.6789	0.0626	-0.3754	0.1802	-0.1134	-0.2769	0.4964	0.1259	-0.1542	0.2690	-0.6122
PC8	42.0694	1.7698	0.8987	0.0120	-0.1452	-0.1049	0.0705	0.0940	-0.3282	0.1320	-0.5988	0.6549	0.2035
PC9	34.4061	1.1838	0.8237	0.2773	-0.2446	0.3446	-0.1519	-0.1997	-0.6428	0.1355	0.4546	0.1516	-0.1431
PC10	12.4241	0.1544	0.6556	0.0309	0.0242	-0.0980	0.0714	0.0098	0.0997	-0.8024	0.3133	0.4813	-0.0098

p<0.001*** p<0.01** p<0.05*

Table S9 Principal Component Analysis statistics and loadings values for the paired comparison Flake “Hold” vs Flake “Aim” (23 participants). Selection of channels based on Wilcoxon signed-rank test. Significant p-values are highlighted in bold. Muscle abbreviations: FCR= *flexor carpi radialis*; TE= thenar eminence muscle group; DI1= first dorsal *interosseus*; ndDI1= non-dominant first dorsal *interosseus*; ndTE= non-dominant thenar eminence group. All other channels correspond to EEG electrodes following the 10-20 international system (32-channel configuration).

**Flake Hold vs Flake Aim**													
				**PCA Loadings**									
	**Eigenvalues**	**% Variance**	**Wilcoxon test PC scores (p-value)**	**F3**	**CP5**	**C4**	**FC5**	**T7**	**ndTE**	**ndDI1**	**FCR**	**TE**	**DI1**
PC1	223.3091	49.8669	**2.38E-07*****	0.2615	0.3308	-0.3274	0.3013	0.3442	0.3963	0.4006	-0.3097	-0.2308	0.1976
PC2	120.3708	14.4891	0.4274	0.5301	0.2378	0.0649	0.5190	-0.0374	-0.2356	-0.2192	0.2745	0.4357	0.1373
PC3	106.7882	11.4037	0.9168	0.1728	0.2227	0.0761	0.0728	0.2488	-0.0523	-0.1333	0.3050	-0.4705	-0.7160
PC4	89.4394	7.9994	0.6221	0.3815	-0.5015	-0.3642	0.1825	-0.5421	0.0386	0.0748	-0.1932	-0.1298	-0.2862
PC5	76.4806	5.8493	0.7090	0.2077	-0.0582	0.7029	0.0844	0.0545	-0.1622	0.0982	-0.6302	-0.0233	-0.1253
PC6	65.7230	4.3195	0.7768	0.0234	-0.1756	0.2957	0.2450	-0.1517	0.1765	-0.3015	0.2058	-0.6234	0.4951
PC7	49.8758	2.4876	0.7090	0.1145	-0.5889	0.1177	0.0516	0.5142	0.4812	-0.1267	0.1404	0.2863	-0.0990
PC8	42.1526	1.7768	0.3146	0.6321	0.0642	-0.0471	-0.7193	0.0662	-0.0064	-0.1600	-0.0009	-0.0810	0.2005
PC9	32.6962	1.0690	0.6869	0.0846	0.2133	0.3837	-0.1183	-0.4347	0.5263	0.4203	0.3293	0.1507	-0.1110
PC10	27.1760	0.7385	0.9406	0.1084	-0.3199	0.0730	-0.0062	0.2014	-0.4660	0.6703	0.3617	-0.1354	0.1561

p<0.001*** p<0.01** p<0.05*

Table S10 Principal Component Analysis statistics and loadings values for the paired comparison Hammerstone "Hold" vs Hammerstone "Aim" (22 participants). Selection of channels based on Wilcoxon signed-rank test. Significant p-values are highlighted in bold. Muscle abbreviations: FCR= *flexor carpi radialis*; HTE= hypothenar eminence muscle group; DI1= first dorsal *interosseus*; ndDI1= non-dominant first dorsal *interosseus*; ndTE= non-dominant thenar eminence group. All other channels correspond to EEG electrodes following the 10-20 international system (32-channel configuration).

Hammerstone Hold vs Hammerstone Aim

				PCA Loadings									
	**Eigenvalues**	**% Variance**	**Wilcoxon test PC scores (p-value)**	**F3**	**FC1**	**C3**	**CP5**	**Fz**	**ndTE**	**ndDI1**	**FCR**	**HTE**	**DI1**
PC1	212.5915	45.1952	**2.38E-06*****	0.2488	0.4287	0.4027	0.3818	0.3697	0.3413	0.2509	0.2944	-0.0839	0.1912
PC2	135.9379	18.4791	0.1289	0.3524	0.0748	0.1025	0.0112	-0.1001	-0.4082	-0.3518	0.4049	0.6074	0.1622
PC3	111.4787	12.4275	0.6556	0.3625	0.2294	0.2393	0.1452	-0.0259	-0.1658	-0.0933	-0.3647	-0.0070	-0.7535
PC4	102.5033	10.5069	0.4060	0.4746	-0.0145	-0.1298	-0.2423	-0.3731	0.2145	0.6189	-0.1869	0.2884	0.1128
PC5	73.5551	5.4104	0.9240	0.1607	-0.2753	0.2955	0.4438	-0.5906	-0.1736	-0.0449	-0.0120	-0.4151	0.2465
PC6	58.5187	3.4244	0.5661	0.3471	0.2083	-0.0548	-0.5708	-0.1387	0.1231	-0.2604	0.3591	-0.5226	-0.0560
PC7	43.3337	1.8778	0.7502	0.2868	-0.0070	-0.7277	0.3849	0.1957	-0.2859	0.1696	0.1926	-0.2195	-0.0771
PC8	38.2483	1.4629	0.7990	0.3131	0.0844	-0.2249	0.1189	0.1155	0.3210	-0.5271	-0.5395	0.0316	0.3793
PC9	28.3601	0.8043	0.9493	0.1006	-0.5153	-0.0735	0.1990	-0.0641	0.6036	-0.1880	0.3352	0.1740	-0.3646
PC10	20.2844	0.4115	0.7745	0.3472	-0.6049	0.2782	-0.2230	0.5382	-0.2213	0.0995	-0.1090	-0.1263	0.1000

p<0.001*** p<0.01** p<0.05*

Table S11 Two-Block Partial Least Squares Analysis. Results for Hold hammerstone vs. Hold flake. The analysis was performed using the 5 EEG channels (block 1) selected based on the highest accuracy in the LDA-based permutation test and all EMG channels (block 2), after PCA outliers' removal. Muscle abbreviations: FCR= *flexor carpi radialis*; FCU= *flexor carpi ulnaris*; FPL= *flexor pollicis longus*; TE= thenar eminence muscle group; HTE= hypothenar eminence muscle group; DI1= first dorsal *interosseus*. All other channels correspond to EEG electrodes following the 10-20 international system (32-channel configuration).

Hold vs Hold

							**PLS axis 1**
**EEG Block**	**O2**	**Oz**	**CP6**	**F3**	**CP5**		
neg_x	2.46410	2.24848	0.93302	-1.61658	1.40379		
pos_x	-2.46410	-2.24848	-0.93302	1.61658	-1.40379		
**EMG Block**	**FCR**	**HTE**	**FCU**	**FPL**	**DI1**	**TE**	
neg_y	3.00071	2.60330	2.45166	3.24790	2.12049	0.33199	
pos_y	-3.00071	-2.60330	-2.45166	-3.24790	-2.12049	-0.33199	

	**Singular value**	**% total covar**	**Corr coefficient**	**p-value**
PLS axis 1	1.717174786	93.04061826	0.6243756	**0.001*******
PLS axis 2	0.370568028	4.332908068	0.470215665	0.178
PLS axis 3	0.21653222	1.479410302	0.42007124	0.117
PLS axis 4	0.183053092	1.057298758	0.291218141	**0.002****
PLS axis 5	0.053337272	0.089764617	0.170139188	0.183

p<0.001*** p<0.01** p<0.05*

Table S12 Two-Block Partial Least Squares Analysis. Results for Aim hammerstone vs. Aim flake. The analysis was performed using the 5 EEG channels (block 1) selected based on the highest accuracy in the LDA-based permutation test and all EMG channels (block 2), after PCA outliers' removal. Muscle abbreviations: FCR= *flexor carpi radialis*; FCU= *flexor carpi ulnaris*; FPL= *flexor pollicis longus*; TE= thenar eminence muscle group; HTE= hypothenar eminence muscle group; DI1= first dorsal *interosseus*; ndDI1= non-dominant first dorsal *interosseus*; ndTE= non-dominant thenar eminence group. All other channels correspond to EEG electrodes following the 10-20 international system (32-channel configuration).

Aim vs Aim

								PLS axis 1
**EEG Block**	**O2**	**P8**	**P4**	**Oz**	**CP6**			
neg_x	2.361802	2.758415	1.369363	2.128551	0.97026			
pos_x	-2.3618	-2.75842	-1.36936	-2.12855	-0.97026			
**EMG Block**	**TE**	**FCU**	**FPL**	**HTE**	**DI1**	**FCR**	**ndTE**	**ndDI1**
neg_y	1.075946	1.009233	2.583892	2.640942	1.999408	2.141022	-1.45654	-2.1377
pos_y	-1.07595	-1.00923	-2.58389	-2.64094	-1.99941	-2.14102	1.456543	2.137704

	Singular value	% total covar	Corr coefficient	p-value
PLS axis 1	1.361638	82.25814	0.484935	**0.028***
PLS axis 2	0.392236	6.825763	0.454537	0.576
PLS axis 3	0.363292	5.855559	0.573667	**0.025***
PLS axis 4	0.287854	3.676218	0.378151	**0.001*****
PLS axis 5	0.17664	1.384317	0.245638	**0.001*****

p<0.001*** p<0.01** p<0.05*

Table S13 Two-Block Partial Least Squares Analysis. Results for Hold vs. Aim in flake use. The analysis was performed using the 5 EEG channels (block 1) selected based on the highest accuracy in the LDA-based permutation test and all EMG channels (block 2). Muscle abbreviations: FCR= *flexor carpi radialis*; FCU= *flexor carpi ulnaris*; FPL= *flexor pollicis longus*; TE= thenar eminence muscle group; HTE= hypothenar eminence muscle group; DI1= first dorsal *interosseus*; ndDI1= non-dominant first dorsal *interosseus*; ndTE= non-dominant thenar eminence group. All other channels correspond to EEG electrodes following the 10-20 international system (32-channel configuration).

F-hold vs F-aim

PLS axis 1

**EEG Block**	**C3**	**C4**	**CP1**	**FC1**	**F3**			
neg_x	-2.49201	3.335013	-0.70592	-1.21568	-1.6252			
pos_x	2.492014	-3.33501	0.705916	1.215675	1.625197			
**EMG Block**	**ndTE**	**ndDI1**	**TE**	**DI1**	**FCU**	**FCR**	**FPL**	**HTE**
neg_y	-3.18902	-2.60416	1.326051	-1.6259	-1.39471	1.310167	-1.0065	-0.08291
pos_y	3.189022	2.604158	-1.32605	1.625898	1.394714	-1.31017	1.0065	0.082907

	**Singular value**	**% total covar**	**Corr coefficient**	**p-value**
PLS axis 1	1.707769	84.63484	0.642947	**0.001*** **
PLS axis 2	0.596744	10.33398	0.573851	**0.03***
PLS axis 3	0.386072	4.325408	0.32917	**0.006****
PLS axis 4	0.133358	0.516091	0.286871	0.373
PLS axis 5	0.080849	0.189689	0.287781	0.052

p<0.001*** p<0.01** p<0.05*

Table S14 Two-Block Partial Least Squares Analysis. Results for Hold vs. Aim in hammerstone use. The analysis was performed using the 5 EEG channels (block 1) selected based on the highest accuracy in the LDA-based permutation test and all EMG channels (block 2), after PCA outliers' removal. Muscle abbreviations: FCR= *flexor carpi radialis*; FCU= *flexor carpi ulnaris*; FPL= *flexor pollicis longus*; TE= thenar eminence muscle group; HTE= hypothenar eminence muscle group; DI1= first dorsal *interosseus*; ndDI1= non-dominant first dorsal *interosseus*; ndTE= non-dominant thenar eminence group. All other channels correspond to EEG electrodes following the 10-20 international system (32-channel configuration).

**H-Hold vs H-Aim**								
								**PLS axis 1**
**EEG Block**	**P7**	**C4**	**FC5**	**F3**	**FC1**			
neg_x	-2.27251	0.337711	-2.38789	-2.37706	-3.0514			
pos_x	2.272512	-0.33771	2.387889	2.377062	3.051397			
**EMG Block**	**ndTE**	**ndDI1**	**FCU**	**DI1**	**TE**	**FCR**	**HTE**	**FPL**
neg_y	-2.70181	-3.23609	-0.99815	-1.60646	-0.65426	-1.72508	1.006897	-0.16483
pos_y	2.701806	3.236087	0.998146	1.606458	0.654259	1.725078	-1.0069	0.164826

	**Singular value**	**% total covar**	**Corr coefficient**	**p-value**
PLS axis 1	2.077953	92.78287	0.722968	**0.001*****
PLS axis 2	0.484194	5.037724	0.737573	0.277
PLS axis 3	0.274957	1.624519	0.319573	0.253
PLS axis 4	0.122036	0.320015	0.140708	0.535
PLS axis 5	0.104549	0.234873	0.330351	**0.043***

p<0.001*** p<0.01** p<0.05*
